# Redundancy and modularity in membrane-associated dissimilatory nitrate reduction in *Bacillus*

**DOI:** 10.3389/fmicb.2012.00371

**Published:** 2012-10-18

**Authors:** Kim Heylen, Jan Keltjens

**Affiliations:** ^1^Laboratory of Microbiology, Department of Biochemistry and Microbiology, University of GhentGent, Belgium; ^2^Department of Microbiology, IWWR, Radboud University NijmegenNijmegen, Netherlands

**Keywords:** dissimilatory nitrate reduction to ammonium (DNRA), denitrification, ammonification, nitric oxide reductase

## Abstract

The genomes of two phenotypically denitrifying type strains of the genus *Bacillus* were sequenced and the pathways for dissimilatory nitrate reduction were reconstructed. Results suggest that denitrification proceeds in the periplasmic space and in an analogous fashion as in Gram-negative organisms, yet with the participation of proteins that tend to be membrane-bound or membrane-associated. A considerable degree of functional redundancy was observed with marked differences between *B. azotoformans* LMG 9581^T^ and *B. bataviensis* LMG 21833^T^. In addition to the already characterized menaquinol/cyt *c*-dependent nitric oxide reductase (Suharti et al., 2001, 2004) of which the encoding genes could be identified now, evidence for another novel nitric oxide reductase (NOR) was found. Also, our analyses confirm earlier findings on branched electron transfer with both menaquinol and cytochrome *c* as reductants. Quite unexpectedly, both bacilli have the disposal of two parallel pathways for nitrite reduction enabling a life style as a denitrifier and as an ammonifying bacterium.

## Introduction

Nitrogen is an essential element of all forms of life, but most of it on Earth is only available in an inaccessible form as dinitrogen gas (N_2_). Nitrogen-fixing microorganisms, however, are able to bind N_2_ into ammonium, which serves as a nutrient for other organisms. Nitrifiers use ammonium as the electron donor in their energy metabolism by oxidizing it to nitrite and nitrate. The latter two compounds are utilized by other microbial species for anaerobic respiration, reducing nitrite and nitrate back to dinitrogen gas in a process called denitrification or dissimilatory nitrate/nitrite reduction, thus closing the biogeochemical nitrogen cycle (N-cycle). These three basic processes have been known for over hundred years, but in the last decades it has become clear that the N-cycle is definitely more complex. It now appears that two major processes have been overlooked for a long time: anaerobic ammonium oxidation (anammox) and dissimilatory reduction of nitrite/nitrate into ammonium (DNRA), also known as nitrate/nitrite ammonification [see for recent reviews: Jetten (2008); Kraft et al. (2011); Lam and Kuypers (2011); Martínez-Espinosa et al. (2011)]. Like classical denitrifiers, DNRA bacteria employ nitrite and nitrate as electron acceptors for respiration (Einsle et al., 2002). They, however, reduce these compounds to ammonium as the name suggests. Hitherto, no species are known that carry out both denitrification and DNRA.

In its canonical form, dissimilatory nitrate reduction consists of four consecutive steps: (1) reduction of nitrate to nitrite, (2) nitrite reduction to nitric oxide (NO), (3) NO reduction to produce nitrous oxide (N_2_O), and (4) reduction of the latter to N_2_. The specific enzyme for N_2_O reduction, N_2_O reductase (NOS, N_2_OR) is not always present and microorganisms lacking this enzyme make N_2_O as their end product. In practice, a “true denitrifier” converts at least 80% of the consumed nitrate or nitrite to either N_2_O or N_2_ (Mahne and Tiedje, 1995). Also DNRA bacteria are capable of N_2_O formation, albeit in non-stoichiometric amounts that don't exceed 2–36% of consumed nitrate (Bleakley and Tiedje, 1982; Streminíska et al., 2012). Moreover, the production occurs only during the stationary phase, suggesting emission to be the result of a secondary metabolism (Smith, 1983).

The process of dissimilatory nitrate reduction has been extensively investigated at the genetic, enzymological, and regulatory levels using, among others, *Paracoccus denitrificans*, *Pseudomonas stutzeri*, *Eschericha coli*, and *Wolinella succinogenes* as model organisms [see for example: Tucker et al. (2005); van Wonderen et al. (2008); Bergaust et al. (2010); Pomowski et al. (2011); Peña et al. (2012), and references in Zumft (1997); Kern and Simon (2009); Simon and Klotz (2012)], of which most belong to Gram-negative *Proteobacteria*. In the Gram-negative bacteria, the enzymatic reactions involved in denitrification reside at the periplasm, except for nitrate reduction by the Nar-type nitrate reducase, and are catalyzed either by soluble enzymes (periplasmic nitrate-, nitrite-, and N_2_O reductases) or enzymes having their catalytic site embedded in the membrane (NO reductase). Similarly, electron transfer processes that are related to the different reduction reactions are mediated by a broad variety of soluble cytochrome *c* type or cupredoxin-like copper proteins localized in the periplasm.

Dissimilatory nitrate reduction is not restricted to Gram-negative species. In fact, denitrification and DNRA activities have been observed in a wide species and niche diversity among Gram-positive microorganisms as well, in particular *Bacillus* species (de Barjac and Bonnefoi, 1972; Garcia, 1977; Pichinoty et al., 1978, 1983; Tiedje, 1988; Denariaz et al., 1989). However, our knowledge at the molecular and genomic levels on this taxonomic lineage is rather fragmentary. Whereas many genomes of the representatives of the genus *Bacillus* are available, only four genomes have been reported to contain genes encoding denitrification or ammonification key enzymes. The genome of *Bacillus selenitireducens* was found to harbor a homolog of the gene coding for dissimilatory nitrite reductase making ammonium (*nrfA*), while *qnorB* homologs, encoding the quinol-dependent nitric oxide reductase (NOR), were detected in *Bacillus coagulans* XZL4 (Su et al., 2011), *Bacillus licheniformis* ATCC 14580^T^ (Rey et al., 2004; Veith et al., 2004), and *Bacillus* sp. BTB_CT2. None of these species can be unequivocally designated as denitrifiers. Unlike Gram-negative bacteria, Gram-positive microorganisms only have a very limited periplasmic space. In this respect, it is remarkable that activities of denitrifying enzymes were consistently associated with membrane fractions (Denariaz et al., 1991; Urata and Satoh, 1991; Suharti et al., 2001; Suharti and de Vries, 2005; Fukuda et al., 2011; Matsumoto et al., 2012). Similarly, the limited number of such enzymes that have been purified to date from bacilli or close relatives are all membrane proteins (Denariaz et al., 1991; Urata and Satoh, 1991; Suharti et al., 2001; Suharti and de Vries, 2005; Fukuda et al., 2011; Matsumoto et al., 2012).

Considering the wide-spread occurrence of dissimilatory nitrate reduction and DNRA among *Bacillus* species (Verbaendert et al., 2011), their ecological relevance, niche, and species differentiation, possibly particular demands that are posed to the localization and organization of enzymes involved in these processes, and an overall limited knowledge regarding their molecular mechanisms, we now sequenced the genomes of two well-known denitrifying, publicly available representatives, *Bacillus azotoformans* LMG 9581^T^ (Pichinoty et al., 1983) and *Bacillus bataviensis* LMG 21833^T^ (Heyrman et al., 2004), able to produce dinitrogen and nitrous oxide respectively as end-products (Verbaendert et al., unpublished data). In order to come to a more comprehensive understanding of nitrate respiration by these Gram-positive microorganisms, we focused on their denitrifying inventories. Using bio-informatic tools, all gene products were analyzed for their homology with well studied enzymes from other organisms, their cellular localization and, in case of membrane proteins, for their putative topology and orientation. The results of our study enabled an unprecedented insight into the genomic basis underlying denitrification in both *Bacillus* species.

## Materials and methods

### Strains and DNA extraction

*Bacillus azotoformans* LMG 9581^T^ and *Bacillus bataviensis* LMG 21833^T^ were obtained from the BCCM/LMG bacteria collection. Strains were aerobically grown on trypticase soy broth at 28°C. Cells were harvested after overnight growth and DNA was extracted by the method of Pitcher et al. (1989), slightly modified as described previously (Heyndrickx et al., 1996).

### Genome sequencing

Library preparation and genome sequencing was performed by Baseclear B.V. For sequencing, a paired-end strategy on the Illumina Genome Analyzer IIx was used that yielded reads of an average length of 74 bp. Automatic trimming (based on a threshold of *Q* = 20) and assembly was performed using CLC Genomics Workbench v4. The k-mer parameter was varied to maximize the N50 of the resulting assembly for each genome. Genome statistics are listed in Table 1.

**Table 1:** **Genome characteristics of both analyzed genomes**.

	*B. azotoformans* LMG 9581^T^	*B. bataviensis* LMG 21833^T^
# contigs	169	197
Size (Mb)	4,3 MB	5,4 Mb
Av. read coverage	86x	79,5x
N50 (Kb)	94,5	82,1
% G+C	39,7	39,6
# RNA calls	8 rRNA	6 rRNA
	25 tRNA	23 tRNA
# CDS calls	4226	5207
NCBI accession n°	AJLR00000000	AJLS00000000
NCBI BioProject	PRJNA80827	PRJNA77725

Calls for RNA and CDS were deduced from PGAAP.

### Genome annotation

Functional annotation and metabolic reconstruction was performed with (1) the Rapid Annotation Subsystem Technology (RAST) server (Aziz et al., 2008), using Glimmer (Salzberg et al., 1998) for gene calling and allowing frameshift correction, backfilling of gaps, and automatic fixing of errors, (2) KEGG Automatic Annotation Server (KAAS) (Moriya et al., 2007), using Glimmer gene calls from RAST and total prokaryotic genes data set for annotation, and (3) NCBI's Prokaryotic Genome Automatic Annotation Pipeline (PGAAP) (http://www.ncbi.nlm.nih.gov/genomes/static/Pipeline.html) which uses GeneMark and GeneMark.HMM for gene calling (Borodovsky and McIninch, 1993; Lukashin and Borodovsky, 1998). Assigned functions were checked with pBLAST (Altschul et al., 1997) and InterProScan (Zdobnov and Apweiler, 2001). An inventory of genes involved in denitrification and ammonification for both genomes are listed in Tables 2, 3. Missing genes were searched for in the genome with PSI-BLAST using homologous amino acid sequences of closely related *Bacillus* or *Geobacillus* species. Possible frameshifts mentioned in the text were not corrected in the submitted genome.

**Table 2:** **Overview of gene inventory involved in nitrogen assimilation, denitrification and ammonification of *B. azotoformans* LMG 9581^T^**.

**Function**	**Gene**	**Gene coordinates**	**Size (bp)**	**pBLAST best hit**			**ORF identifier**
							
				**Genbank identifier**	**% ID**	**Rel. gene length**	
Cytoplasmic dissimilatory nitrate reduction	*narG1*	contig48_8658_4975	3684	ABO66033 *Geobacillus thermodenitrificans* NG80-2	73	100,1	BAZO_08891
	*narH1*	contig48_4985_3435	1551	ABO66034.1 *Geobacillus thermodenitrificans* NG80-2	73	101,4	BAZO_08886
	*narJ1*	contig48_3463_2900	564	ABO66035 *Geobacillus thermodenitrificans* NG80-2	46	101,1	BAZO_08881
	*narI1*	contig48_2856_2140	717	YP_001124781.1 *Geobacillus thermodenitrificans* NG80-2	65	103,9	BAZO_08876
	*narG2*	contig69_15227_11544	3684	EEL50916 *Bacillus cereus* Rock3-44	80	98,0	BAZO_10677
	*narH2*	contig69_11554_10088	1467	EID44195 *Geobacillus thermoglucosidans* TNO-09.020	84	99,8	BAZO_10672
	*narJ2*	contig69_10009_9455	555	YP_005422967 *Bacillus amyloliquefaciens* subsp. *plantarum* YAU B9601-Y2	46	100,0	BAZO_10667
	*narI2*	contig69_9458_8769	690	YP_002949597.1 *Geobacillus* sp. WCH70	69	98,3	BAZO_10662
Periplasmic dissimilatory nitrate reduction	*napG1*	contig124_32161_32757	597	ZP_07949106.1 *Eggerthella* sp. 1_3_56FAA	57	97,5	BAZO_15064
	*napA*	contig124_32833_35370	2538	YP_004710008.1 *Eggerthella* sp. YY7918	61	99,3	BAZO_15069
	*napB*	contig124_35383_35802	420	YP_003303796.1 *Sulfurospirillum deleyianum* DSM 6946	28	80,9	BAZO_15074
	*napD*	contig124_35847_36104	258	ZP_07949109.1 *Eggerthella* sp. 1_3_56FAA	38	101,2	BAZO_15079
	*napH*	contig121_9516_10421	906	ZP_07949110.1 *Eggerthella* sp. 1_3_56FAA	40	101,3	BAZO_14359
	*napG2*	contig124_36110_36679	570	ZP_09635522 *Desulfitobacterium dehalogenans* ATCC 51507	36	101,6	BAZO_15084
Assimilatory nitrate reduction	*nasC*						Not present
Assimilatory nitrite reduction	*nirB*						Not present
	*nirD*						Not present
Nitrate transport	*narK*	contig69_15531_17030	1500	ZP_08006147.1 *Bacillus* sp. 2_A_57_CT2	66	99,6	BAZO_10682
Nitrite transport	*nirC1*	contig05_16758_15988	768	ZP_09351718.1 *Bacillus smithii* 7_3_47FAA	62		BAZO_00505
	*nirC2*	contig107_<1_505	505	YP_005876144.1 *Lactococcus lactis* subsp. *cremoris* A76	97		BAZO_11644
Ammonium transport							Not present
Dissimilatory nitrite reduction to ammonium	*nrfH*	contig37_9421_9936	516	YP_002435907.1 *Desulfovibrio vulgaris* str. 'Miyazaki F'	46	108,2	BAZO_03250
	*nrfA*	contig37_9926_11395	1469	ZP_09602448.1 *Bacillus* sp. 1NLA3E	65	103,1	BAZO_03255
Dissimilatory nitrite reduction to NO	*nirK*	contig38_30517_31578	1062	ZP_08007035.1 *Bacillus* sp. 2_A_57_CT2	74	100,3	BAZO_03565
Dissimilatory quinol-dependent nitric oxide reduction	*qnorB1*	contig04_38763_36502	2262	ZP_09601292.1 *Bacillus* sp. 1NLA3E	82	99,3	BAZO_00190
	*qnorB2*	contig48_12741_10378	2364	ZP_09599319.1 *Bacillus* sp. 1NLA3E	77	100,5	BAZO_08916
	*norD/yojO*	contig15_10303_8381	1923	AEN89960.1 *Bacillus megaterium* WSH-002	58	100,5	BAZO_01537
	*norQ/yojN*	contig15_11194_10313	882	YP_003597326.1 *Bacillus megaterium* DSM 319	73	96,7	BAZO_01542
	*dnrN/norA*	contig39_94731_95435	705	ZP_08532570.1 *Caldalkalibacillus thermarum* TA2.A1	56	98,7	BAZO_04395
Dissimilatory menaquinol/cyt *c*-dependent nitric oxide reduction (Type I) (qCu_A_NOR, sNOR)	*cbaC1*	contig42_104893_105033	138	ZP_08784194.1 *Ornithinibacillus scapharcae* TW25	38	102,2	–
	*cbaB1*	contig42_105051_105512	462	YP_004095308.1 *Bacillus cellulosilyticus* DSM 2522	70	99,4	BAZO_06394
	*cbaA1*	contig42_105527_107233	1707	ZP_08532586.1 *Caldalkalibacillus thermarum* TA2.A1	71	100,9	BAZO_06399
	*senC1*	contig42_107320_107955	636	YP_004095310.1 *Bacillus cellulosilyticus* DSM	62	100,5	BAZO_06404
	*cbaD1*	contig42_107955_108527	570	ZP_08008054.1 *Bacillus* sp. 2_A_57_CT2	69	102.1	BAZO_06409
Putative dissimilatory menaquinol/cyt *c*-dependent nitric oxide reduction (Type II)	*cbaC2*	contig40_60717_60133	582	ZP_03146936.1 *Geobacillus* sp. G11MC16	44	101.0	BAZO_04695
	*senC2*	contig40_61349_60744	606	ABO66889.1 *Geobacillus thermodenitrificans* NG80-2	44	95,7	BAZO_04700
	*cbaA2*	contig40_62823_61342	1482	ABO66888.1 *Geobacillus thermodenitrificans* NG80-2	65	102,5	BAZO_04705
	*cbaB2*	contig40_63368_62850	519	ZP_03146939.1 *Geobacillus* sp. G11MC16	58	96,6	BAZO_04710
Dissimilatory nitrous oxide reduction	*nosF0*	contig04_11581_12357	777	ZP_07028021.1 *Afipia* sp. 1NLS2	52	101,2	BAZO_00080
	*nosC1*	contig04_19964_20425	459	ZP_08982140.1 *Desulfosporosinus meridiei* DSM 13257	36	104,1	BAZO_00115
	*nosZ1*	contig04_20450_22300	1851	YP_00454079.1 *Desulfotomaculum ruminis* DSM 2154	66	97,3	BAZO_00120
	*nosD1*	contig04_22977_24377	1401	AEG59795.1 *Desulfotomaculum ruminis* DSM 2154	36	95,3	BAZO_00130
	*nosL1*	contig04_24398_24883	485	ZP_09074493.1 *Paenibacillus elgii* B69	36	83,8	BAZO_00135
	*nosY1*	contig04_24858_25700	843	YP_002456717.1 *Desulfitobacterium hafniense* DCB-2	45	101,1	BAZO_00140
	*nosF1*	contig04_25697_26614	918	ZP_09636992.1 *Desulfitobacterium dehalogenans*	43	100,0	BAZO_00145
	*nosC2*	contig41_6372_6797	423	YP_001125844.1 *Geobacillus thermodenitrificans* NG80-2	48	93,4	BAZO_05335
	*nosZ2*	contig41_6822_8702	1881	YP_001125843.1 *Geobacillus thermodenitrificans* NG80-2	76	100,8	BAZO_05340
	*nosD2*	contig41_9319_10644	1326	YP_001125841.1 *Geobacillus thermodenitrificans* NG80-2	48	102,1	BAZO_05350
	*nosC3*	contig147_57854_58288	432	YP_001125844.1 *Geobacillus thermodenitrificans* NG80-2	51	95,4	BAZO_18221
	*nosZ3*	contig147_58304_60181	1878	YP_001125843.1 *Geobacillus thermodenitrificans* NG80-2	78	100,6	BAZO_18226
	*nosD3*	contig147_60810_62126	1317	YP_001125841.1 *Geobacillus thermodenitrificans* NG80-2	55	101,4	BAZO_18236
	*nosY3*	contig147_62126_62923	798	YP_001125840.1 *Geobacillus thermodenitrificans* NG80-2	48	100,4	BAZO_18241
	*nosF3*	contig147_62920_63591	672	YP_001125839.1 *Geobacillus thermodenitrificans* NG80-2	65	101,8	BAZO_18246
	*nosD4*	contig147_26847_28190	1344	YP_002315691.1 *Anoxybacillus flavithermus* DSM 21510 / WK1	37	100,7	BAZO_18066
	*nosL4*	contig147_28190_28672	482	YP_002315692.1 *Anoxybacillus flavithermus* DSM 21510 / WK1	45	108,6	BAZO_18071
	*nosY4*	contig147_28713_29543	831	YP_002315693.1 *Anoxybacillus flavithermus* DSM 21510 / WK1	43	103,0	BAZO_18076
	*nosF4*	contig147_29548_30279	732	YP_002315694.1 *Anoxybacillus flavithermus* DSM 21510 / WK1	53	104,7	BAZO_18081
	*nosD5*	contig120_85053_86486	1434	YP_003570259.1 *Salinibacter ruber* M8	28	102,8	BAZO_14249
	*nosL5*	contig120_86476_86982	506	ZP_08007037.1 *Bacillus* sp. 2_A_57_CT2	41	107,4	BAZO_14254
	*nosY5*	contig120_87020_87985	966	ZP_08007038.1 *Bacillus* sp. 2_A_57_CT2	52	115,4	BAZO_14259
SenC/SCO_1_-type membrane-bound protein	*senC3*	contig09_37800_37213	522	YP_002316585.1 *Anoxybacillus flavithermus* WK1	55	88,8	BAZO_01207
	*senC4*	contig147_56968_57558	591	ZP_01173458.1 *Bacillus* sp. NRRL B-14911	53	101,5	BAZO_18216

Genes are ordered according to their location in the operon when applicable. Gene size is given in absolute numbers as well as relative to that of best pBLAST hit. No ORF identifier is given when ORF was too small to be recognized in initial annotation.

**Table 3:** **Overview of gene inventory involved in nitrogen assimilation, denitrification and ammonification of *B. bataviensis* LMG 21833^T^**.

**Function**	**Gene**	**Gene coordinates**	**Size (bp)**	**pBLAST best hit**			**ORF identifier**
							
				**Genbank identifier**	**% ID**	**Rel. gene length**	
Cytoplasmic dissimilatory nitrate reduction	*narG*	contig126_61985_58326^a^	3660	YP_001124778.1 *Geobacillus thermodenitrificans* NG80-2	73	99,4	BABA_18597
	*narH*	contig126_58336_56810	1527	YP_001124779.1 *Geobacillus thermodenitrificans* NG80-2	82	99,8	BABA_18592
	*narJ*	contig126_56817_56287	531	YP_001124780.1 *Geobacillus thermodenitrificans* NG80-2	44	95,2	BABA_18587
	*narI*	contig126_56275_55547	729	YP_001124781.1 *Geobacillus thermodenitrificans* NG80-2	66	105,7	BABA_18582
Periplasmic dissimilatory nitrate reduction	*napA*						not present
	*napB*						not present
	*napD*						not present
	*napH*	contig114_85875_86333	459	AEN91893.1 *Bacillus megaterium* WSH-002	41	90,0	BABA_14547
	*napG*						not present
Assimilatory nitrate reduction	*nasC*	contig121_7018_4829	2190	YP_005311789.1 *Paenibacillus mucilaginosus* 3016	80	99,5	BABA_16882
Assimilatory nitrite reduction	*nirB*	contig05_23966_21540	2427	ZP_09602179.1 *Bacillus* sp. 1NLA3E	75	100,1	BABA_00915
	*nirD*	contig05_21543_21223	321	ZP_09602178.1 *Bacillus* sp. 1NLA3E	60	99,1	BABA_00910
Nitrate transport	*narK1*	contig115_127063_125732	1332	YP_003562112.1 *Bacillus megaterium* QM B1551	84	98,4	BABA_15587
	*narK2*	contig126_45914_44613	1302	YP_004643043.1 *Paenibacillus mucilaginosus* KNP414	78	99,5	BABA_18532
	*narK3*	contig126_47642_46140	1503	YP_002774204.1 *Brevibacillus brevis* NBRC 100599	65	100,4	BABA_18537
	*narK4*	contig135_17368_16046	1323	ZP_08680547.1 *Sporosarcina newyorkensis* 2681	68	99,8	BABA_20711
Nitrite transport	*nirC*	contig74_20117_19245	816	ZP_09600582.1 *Bacillus* sp. 1NLA3E	73	98,6	BABA_12510
Ammonium transport	*amt*	contig121_78501_79763	561	ZP_10130407.1 *Bacillus methanolicus* PB1	87	97,4	BABA_17227
Dissimilatory nitrite reduction to ammonium	*nrfA*	contig123_39386_40837	1452	ZP_09602448.1 *Bacillus* sp. 1NLA3E	74	102,1	BABA_17657
	*nrfH*	contig123_38870_39382	513	ZP_09602447.1 *Bacillus* sp. 1NLA3E	55	98,8	BABA_17652
Dissimilatory nitrite reduction to NO	*nirK*	contig42_38340_38029; contig42_38025_37288	1053	ZP_08007035.1 *Bacillus* sp. 2_A_57_CT2	71	99,4	BABA_p06582
Dissimilatory quinol-dependent nitric oxide reduction	*qnorB1*	contig121_76393_77409	1017	ZP_09601292.1 *Bacillus* sp. 1NLA3E	79	44,7	BABA_17212
	*qnorB2*	contig55_76161_75334; contig55_77043_76291	1707	ZP_09599319.1 *Bacillus* sp. 1NLA3E	77	72,5	BABA_p08977
	*norD*						not present
	*norQ*						not present
	*dnrN/norA*	contig134_9390_8692	699	ZP_09599666.1 *Bacillus* sp. 1NLA3E	65	100,9	BABA_20471
Dissimilatory menaquinol/cyt *c*-dependent nitric oxide reduction (Type I) (qCu_A_NOR, sNOR)	*cbaD1*	contig02_44305_43748	570	ZP_08532584.1 *Caldalkalibacillus thermarum* TA2.A1	62	95,9	BABA_00215
	*senC1*	contig02_44940_44308	633	YP_004095310.1 *Bacillus cellulosilyticus* DSM 2522	65	100,0	BABA_00220
	*cbaA1*	contig02_46749_45067	1683	ZP_08008056.1 *Bacillus* sp. 2_A_57_CT2	82	99,5	BABA_00225
	*cbaB1*	contig02_47231_46764	468	ZP_08008055.1 *Bacillus* sp. 2_A_57_CT2	66	74,3	BABA_00230
	*cbaC1*	contig02_47384_47244	138	YP_004095307.1 *Bacillus cellulosilyticus* DSM 2522	46	92,0	–
	*ctaB*	contig02_48671_47742	927	ZP_9916061.1 *Lentibacillus* sp. Grbi	64	99,4	BABA_00235
Putative dissimilatory menaquinol/cyt *c*-dependent nitric oxide reduction (Type II)	*cbaB2*	contig126_34202_34621	420	ZP_08007586.1 *Bacillus* sp. 2_A_57_CT2	66	76,5	BABA_18497
	*ccaA2*	contig126_34739_36205	1467	ZP_08007585.1 *Bacillus* sp. 2_A_57_CT2	74	100,2	BABA_18502
SenC/SCO_1_-type membrane-bound protein	*senC2*	contig36_3182_4066	885	YP_823233.1 bacterium Ellin514	28	96,1	BABA_04124
	*senC3*	contig55_18362_17802	561	EIJ83190.1 *Bacillus methanolicus* MGA3	54	97,4	BABA_08661
	*senC4*	contig126_63835_64437	603	ZP_10131691.1 *Bacillus methanolicus* PB1	78	58,9	BABA_18617

Genes are ordered according to their location in the operon when applicable. Gene size is given in absolute numbers as well as relative to that of best pBLAST hit. No ORF identifier is given when ORF was too small to be recognized in initial annotation.

a The startcodon of BABA_18597 as deposited at DDBJ/EMBL/GenBank, contains a false start codon with an extra 30-bp sequence at the 5′ terminus.

Location of proteins in cells was initially predicted with sequence-based tools as described by Emanuelsson et al. (2007): SignalP 4.0 (Petersen et al., 2011) was used for prediction of secretory signal proteins, TatP 1.0 for twin-arginine translocation signal proteins (Bendtsen et al., 2005), TMHMM 2.0 for transmembrane α-helices (Krogh et al., 2001), LipoP 1.0 (Rahman et al., 2008) for lipoprotein signal proteins, and SecretomeP 2.0 for signal peptide-less secretion (Bendtsen et al., 2004). If a transmembrane helix was predicted in the same region as a signal peptide, results were verified with Phobius (Käll et al., 2004). Whenever available, predictions were checked by comparison to homologous proteins with validated function and resolved crystal structure.

Alignment and phylogenetic analysis were performed with ClustalW 2.0 (Larkin et al., 2007) and MEGA 5.0 (Tamura et al., 2011).

### Accession numbers

The Whole Genome Shotgun projects of *Bacillus azotoformans* LMG 9581^T^ and *Bacillus bataviensis* LMG 21833^T^ have been deposited at DDBJ/EMBL/GenBank under the accession numbers AJLR00000000 and AJLS00000000, respectively. The versions described in this paper are the first versions, AJLR01000000 and AJLS01000000.

## Results and discussion

*Bacillus azotoformans* was originally isolated from garden soil and the organism has been known for decades to be a vigorous denitrifier (Pichinoty et al., 1983). Several biochemical studies investigating the enzymes involved in denitrification were conducted but no genomic data was available to aid interpretation of observations. Therefore, the genome of the type strain of the species was sequenced and the genes associated with dissimilatory nitrate reduction were analyzed. Unexpectedly, the genome contained the genes encoding both the complete denitrification pathway and nitrite ammonification (Table 2). No other organism is known to possess both pathways and also no experimental data has been reported that suggested their concurrence in one organism. This, however, may not be exceptional. Next to *Bacillus azotoformans* LMG 9581^T^ we sequenced the genome of the type strain of *B. bataviensis* LMG 21833^T^, originally isolated from soil (Heyrman et al., 2004). This strain was arbitrarily chosen, but it also contained the gene inventory for both processes (Table 3). Hereafter, we will discuss denitrification and ammonification in *B. azotoformans* step by step, after which both processes will be described for *B. bataviensis* with an emphasis on the differences between both species.

### Nitrate reduction

**Figure 1:**
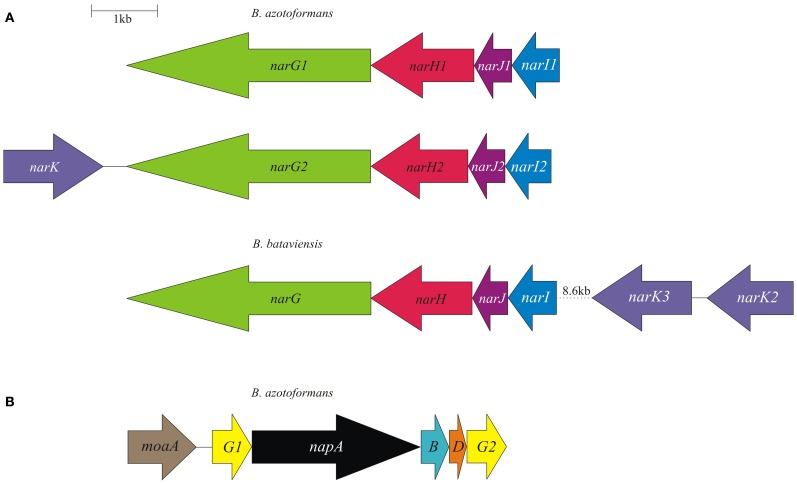
**Physical map of the *B. azotoformans* LMG 9581^T^ and *B. bataviensis* LMG 21833^T^*nar* (A) and *nap* (B) gene clusters.** Arrows show the direction of transcription. Open reading frames are drawn to scale. Homologous genes are shown in identical colors.

The reduction of nitrate to nitrite in Bacteria is catalyzed by three different types of enzymes, that all bind a molybdenum *bis* molybdopterin guanine dinucleotide (Mo-*bis*-MGD) cofactor at the catalytic subunit together with a 4Fe-4S cluster for electron transfer (Rothery et al., 2008). Two of these nitrate reductases (NARs) are involved in respiration, the cytoplasmic Nar, and the periplasmic Nap. The third one, Nas, acts in nitrogen assimilation and is localized in the cytoplasm. In the genome of *B. azotoformans* a gene coding for Nas is absent, but the organism contains the inventory for two functional Nar systems as well as one Nap protein complex (Figure 1). As yet, the presence of a periplasmic NAR has not been reported for a Gram-positive bacterium.

The Nar enzyme is composed of three subunits (NarGHI). In the genome, the genes coding for the three subunits are usually linked with the *narJ* gene encoding a maturation protein. NarGHI is well characterized by the resolution of the atomic structure of the enzyme from *E. coli* (Bertero et al., 2003, 2005). In the complex, NarG is the catalytic subunit housing the Mo-*bis*-MGD cofactor and a 4Fe-4S cluster. In Proteobacteria, NarG and NarH are localized in the cytoplasm, despite the presence in the former of a TAT signal for protein export, but the TAT sequence is non-functional (Ize et al., 2009). NarH binds three 4Fe-4S clusters and one 3Fe-4S cluster in tandem and mediates electron transfer between NarG and NarI. NarI is a membrane protein with five transmembrane helices (TMHs) that sandwich two cytochrome *b* molecules, one near the cytoplasmic and the other one near the periplasmic face. In addition, NarI interacts with the electron donor for nitrate reduction, menaquinol, notably at the periplasmic side (Bertero et al., 2005). The complex is organized such that the uptake of two protons in the cytoplasm during nitrate reduction is associated with the release of two protons at the periplasm upon quinol oxidation, thus contributing to the building of proton-motive force (*pmf*) by a redox loop mechanism (Rothery et al., 2008; Simon et al., 2008).

As mentioned, the genome of *B. azotoformans* codes for two different Nar systems (NarG1H1J1I1, BAZO_08891-08876; NarG2H2J2I2, BAZO_10677-10662) (Figure 1A). In both NarGs (with 74.9% aa sequence identity), all structurally relevant amino acids are fully conserved with respect to those in *E. coli* NarG (data not shown). Similarly, all structurally and functionally relevant amino acids are conserved in both NarH and NarI copies with respect to those of the *E. coli* enzyme. These observations indicate that the two *B. azotoformans* Nars are functional. NarG1 and NarG2 are devoid of a TAT signal sequence, indicative of their cytoplasmic localization. In agreement herewith, the *narG2H2J2I2* gene cluster is linked to a gene coding for a protein (BAZO_10682) of the NarK family of nitrate transporters (Moir and Wood, 2001). More specifically, the pertinent protein is a member of the NarK2 subfamily of nitrate–nitrite antiporters. In other parts of the genome two genes (BAZO_00505, *nirC1*; BAZO_11644, *nirC2*) are found coding for members of the NirC/FocA Major Facilitator Superfamily (MFS). Proteins belonging to this superfamily facilitate the translocation across the cytoplasmic membrane of nitrite (NirC), formate (FocA), or even both (Moir and Wood, 2001; Falke et al., 2010). BAZO_11644 shows 97% sequence identity to *fdhC* gene product of *Lactococcus lactis* subsp. *cremoris* (YP_001032837). FdhC is a formate transporter associated with the formate dehydrogenase, which highly suggests a same function for BAZO_11644. A BlastP search indicates BAZO_00505 to be most closely related to putative formate/nitrite transporters from other *Bacillus* species including *B. subtilis* (NP_390598, 61% aa identity). As compared to FocA and NirC proteins of which the function has been established, sequence similarity of BAZO_00505 is higher with respect to the NirC members, which might imply that this *B. azotoformans* protein favors nitrite export.

The presence of a periplasmic NAR system comes as a surprise. Nature has invented several variations on the Nap theme (Simon and Klotz, 2012) and *B. azotoformans* adds one more. First of all, the *nap* gene cluster organization (*napG1ABDG2-moaA* BAZO_15084-15059) is unusual with two copies of the *napG* gene (*napG1*, BAZO_15064; *napG2*, BAZO_15084) and the presence of a gene (*moaA*; BAZO_15069) coding for one of the enzymes involved in Mo-*bis*-MGD biosynthesis (Table 2; Figure 1B). The *nap* gene cluster is devoid of genes coding for NapH, which is found in another part of the genome (BAZO_14359), whereas coding sequences for the cytoplasmic maturation factors NapF, a 4Fe-4S protein, and NapL are completely absent. Furthermore, no gene accounting for quinol-oxidizing membrane-bound NapC is found on the genome. These genes are also missing in *Campylobacter jejuni*, where *nrfH* seems to replace *napC* (Pittman et al., 2007). Still, the available genes would suffice for an operational Nap system (González et al., 2006). In this system, NapA represents the catalytic subunit harboring a Mo-*bis*-MGD cofactor and a 4Fe-4S cluster like in NarG. NapD is involved in the posttranslational assembly of NapA. NapA receives its electrons for nitrate reduction from the companion diheme *c* protein NapB. NapAB are particularly well characterized by resolution of the crystal structures from at least four different species (Arnoux et al., 2003; Jepson et al., 2007; Kern and Simon, 2008; Najmudin et al., 2008; Coelho et al., 2011). The comparison of the amino acid sequence of *B. azotoformans* NapA with those of known atomic structures reveals the conservation of all amino acids related with the binding of the molybdopterin cofactor and of the iron sulfur cluster. However, NapB contains some specific insertions and deletions with respect to NapB from *Rhodobacter sphaeroides* (Arnoux et al., 2003) and *Cupriavidus necator* (Coelho et al., 2011), possibly indicating somewhat different interactions with other subunits within the enzyme complex, but with conservation of the two heme *c* binding sites (data not shown). *B. azotoformans* NapA contains a distinct TAT signal, whereas NapB has an N-terminal signal sequence, indicating that both proteins are exported to the periplasm, as expected. NapH and one or both NapG proteins most likely constitute a membrane-bound electron-transfer module, as has been established for other organisms (Richardson et al., 2001; Simon et al., 2003; Kern and Simon, 2008). NapH is a membrane-bound enzyme (with four TMHs) that specifically oxidizes menaquinol while NapG is a periplasmic adapter protein that is thought to deliver electrons from menaquinol oxidation (Kern and Simon, 2008). In agreement herewith, NapH from *B. azotoformans* is predicted to contain 4 TMHs, whereas two Cx3CP motifs and the cysteines binding two 4Fe-4S clusters in the periplasmic domain are fully conserved with respect to the NapH proteins from *E. coli* and *P. denitrificans*. Both NapG's are shorter than NapG from *W. succinogenes* (Kern and Simon, 2008), *E. coli* (YP_002403484.1), or *Campylobacter upsaliensis* (ZP_00370550.1) (198 for NapG1 and 189 for NapG2 vs 232-266 aa), lacking a C-terminal part. However, they still contain a 4Fe-4S binding motif (Kern and Simon, 2008) suggesting functionality of both copies. As expected for the periplasmic localization of this iron-sulfur protein, NapG2 (BAZO_15084) has a clear TAT signal sequence, unlike NapG1 (BAZO_15064). Its absence would localize NapG1 in the cytoplasm. It is conceivable that the protein substitutes at this side for NapF found in other organisms. By the presence of the NapAB and NapGH modules, possibly assembled as one membrane-bound complex, *B. azotoformans* has the disposal of a second quinol-dependent NAR system. Presently, it is not understood why *B. azotoformans* as well as many other microorganisms harbor two dissimilatory reductases. By its topology and architecture, Nap is not expected to contribute to the *pmf*. It has been suggested that a main function of Nap would be nitrate-dependent regeneration of quinone from quinol that is produced in concert with the oxidation of the (organic) substrates (Richardson, 2000). Alternatively, the Nap module and the dissimilatory nitrite reductase (NrfHA) discussed next, may provide *B. azotoformans* with a high-affinity system for N assimilation under low nitrate conditions (Pittman et al., 2007; Kim et al., 2012).

### Nitrite reduction

In general, the one-electron reduction of nitrite to NO is catalyzed by two genetically and biochemically distinct nitrite reductases, the cytochrome *cd*_1_ protein (NirS) and copper-containing NirK. *B. azotoformans* contains the latter representative (Table 2). DNRA bacteria possess NrfA as their key enzyme. Assisted by its redox partner NrfH, NrfA catalyzes the six-electron reduction of nitrite to ammonium (Simon, 2002; Einsle, 2011; Simon and Klotz, 2012). NrfHA can also be involved in stress response to nitric oxide, hydroxylamine, and hydrogen peroxide (Kern et al., 2011). *B. azotoformans* contains both NrfA and NrfH. In a similar fashion, the NirB and NirD proteins catalyze the NAD(P)H-dependent reduction of nitrite to ammonium for nitrogen assimilation (Luque-Almagro et al., 2011), but homologs of these cannot be detected in the genome of *B. azotoformans*.

NirK is encoded by BAZO_03565 and the protein shows a high degree of identity with known NirKs of which crystal structures are available (Ellis et al., 2001; Tocheva et al., 2004; Jacobson et al., 2005; Fukuda et al., 2011). Among the structurally well characterized proteins, sequence identity (80%) is highest with NirK (GK0767; YP_146620) from Gram-positive *Geobacillus kaustophilus* (Fukuda et al., 2011). BAZO_03565 shares three characteristic loop regions, with deletions in the “linker loop” and “tower loop,” as well as an “extra loop.” These features are typical for the NirK2 family (Boulanger and Murphy, 2002). The tower loop was suggested to “facilitate a more intimate interaction with the lipid membrane” (Boulanger and Murphy, 2002). BAZO_03565 is preceded by a Sec signal for protein export, indicative of a periplasmic localization of the processed protein. Such localization holds for all NirKs known to date. Quite interestingly, the LipoP program predicts the *B. azotoformans* protein to be a lipoprotein. Such covalent binding confirms results by Suharti and de Vries (2005), who found that nitrite reductase activity of *B. azotoformans* NCCB 10003 was exclusively associated with the membrane fraction. We may note that NirK from randomly chosen Gram-positive bacteria [*Geobacillus thermodenitrificans* NG80-2 (GTNG_0650), *Geobacillus kaustophilus* HTA-426 (GK0767), and *Geobacillus thermoglucosidasius* C56-YS93 (Geoth_3084)] are also identified as putative lipoproteins. Suharti and de Vries (2005) proposed NirK from *B. azotoformans* to be a dual-function enzyme: it could use both menaquinol and reduced cytochrome *c* as electron donors. However, it remained unclear whether menaquinol acted in a direct way, via a menaquinol oxidizing enzyme, or indirectly via the action of menaquinol: cytochrome *c* oxidase (*b*_6_*f*, complex III; see below). Unfortunately, BAZO_03565 takes an isolated position within the genome and the gene context does not give a clue about its redox partners. This is in contrast with observations in the genomes of closely related denitrifiers *Geobacillus thermodenitrificans* (Feng et al., 2007) and *G. kaustophilus*, in which the *nirK* is imbedded in a gene cluster with genes encoding NAR and associated proteins, NOR genes, a nitrate transporter, and several regulatory genes.

**Figure A1:**
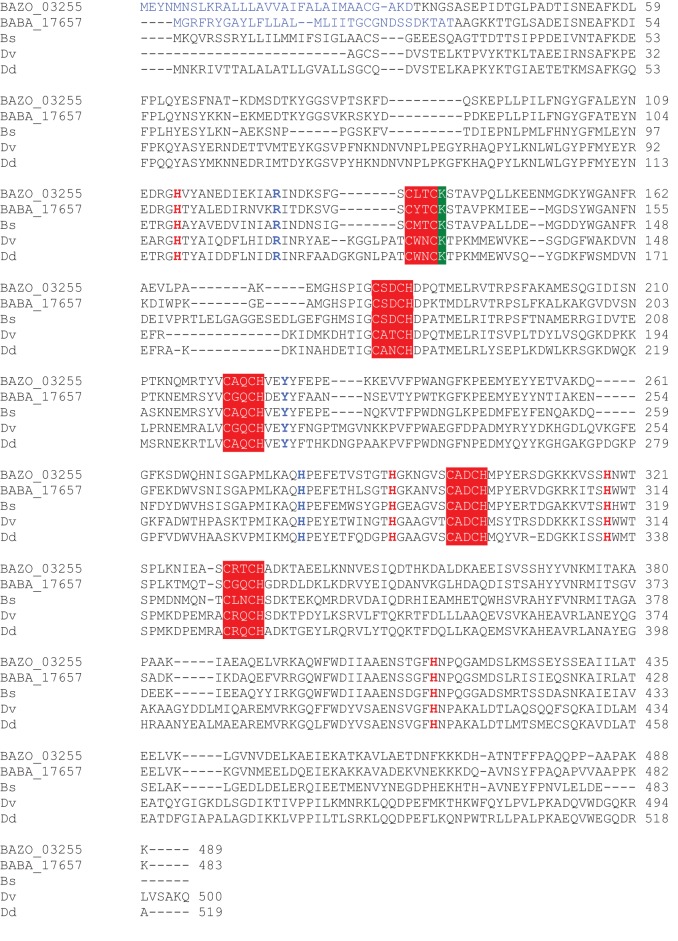
**Multiple sequence alignment of NrfA from *B. azotoformans* LMG 9581^T^, *B. bataviensis* LMG 21833^T^, and other bacteria.** Heme-binding motifs are highlighted in red (white lettering), the lysine at the catalytic heme is highlighted in green, active site residues are printed in blue, distal heme-ligating histidines for hemes 2–6 are printed in red [according to Einsle et al. (2000)]. Bs, *Bacillus selenitireducens* MLS10 (Bsel_1305); Dv, *Desulfovibrio vulgaris* DP4 (PDB 2J7A); Dd, *Desulfovibrio desulfuricans* (PDB 10AH_A). PDB, protein database accession number.

Ammonium-forming nitrite-reducing NrfHA are encoded by BAZO_03250-03255 (Table 2). The *nrfHA* operon organization is similar to that described for several bacteria capable of DNRA such as *Campylobacter jejuni*, *Desulfovibrio vulgaris*, *Geobacter sulfurreducens* (Simon, 2002), and *Desulfitobacterium hafniense* DCB-2 (Kim et al., 2012). NrfA is a periplasmic pentaheme *c* protein in which four cytochromes *c* are involved in electron transfer while the fifth one has a catalytic function. Their presence in the amino acid sequence is characterized by CXXCH (electron transfer) and CXXCK (catalysis) motifs. BAZO_03255, indeed, shows these motifs and it displays also all other sequence features of NrfA proteins of which crystal structures are available (Bamford et al., 2002; Cunha et al., 2003; Rodrigues et al., 2006a) (Figure A1). Consistent with the localization of all known NrfA's, BAZO_03255 contains an N-terminal signal sequence for protein export. Known NrfA's are soluble proteins, but the LipoP program predicts BAZO_03255 to be a lipoprotein. As far as could be checked, the lipoprotein nature might also hold for NrfA's from other *Firmicutes*, namely *Bacillus selenitireducens* MLS10 (Bsel_1305) and *Desulfitobacterium hafniense* DCB-2 (Dhaf_4234). NrfH is a membrane-bound tetraheme cytochrome *c* belonging to the NapC/NirT family of menaquinol oxidases (Simon et al., 2000). The comparison with NrfH from the *Deltaproteobacterium Desulfovibrio vulgaris*, of which the structure has been resolved (Rodrigues et al., 2006b, 2008), establishes the conservation in BAZO_03250 of all relevant amino acids implemented with structuring the N-terminal TMH, quinol binding, and ligation of the four hemes *c*. Taken together, our observations indicate that both NrfA and NrfH are functional proteins in *B. azotoformans*. As a membrane-bound complex, NrfAH would facilitate in *B. azotoformans* menaquinol oxidation coupled with the reduction of nitrite making ammonium. One may note that both proteins are rich in heme *c* molecules. In this respect it is interesting that *nrfAH* is linked to a gene cluster (*ccmEFHABC*; BAZO_03265-BAZO_03300) encoding six out of eight proteins of the System I cytochrome *c* biogenesis machinery (Kranz et al., 2009).

It now appears that *B. azotoformans* has the disposal of two parallel pathways for nitrite reduction enabling a life style as a denitrifier and as a DNRA bacterium. It has been argued that denitrification is more favorable under carbon limitation, whereas as nitrate/nitrite ammonification is more attractive under electron acceptor limitation (Tiedje et al., 1982; Tiedje, 1988). By the presence of the two, *B. azotoformans* may benefit from the best of two worlds. Still, the metabolism leaves us with one puzzle. One may note that the organism is devoid of the assimilatory nitrate and nitrite reductases. This would make *B. azotoformans* dependent on ammonium as the nitrogen source. Obviously, ammonium can be produced by the action of the Nar, Nap, and Nrf systems and even more so, since nrfA in *E. coli* is known to be expressed under low nitrate conditions, while NirB operates at high nitrate concentrations (Wang et al., 2000). So, *B. azotoformans* might have adapted to low nitrate/nitrite conditions. The problem, however, is that no gene is found in the genome of *B. azotoformans* coding for a known (AmtB-type) transporter to take periplasmically produced ammonium into the cell.

### Nitric oxide reduction to nitrous oxide

The product of nitrite reductase NirK, NO, is a very reactive and toxic free radical compound and a range of known or predicted but still to be validated enzymes exists to convert it into N_2_O (Richardson, 2000; de Vries and Schröder, 2002; Tavares et al., 2006; Hemp and Gennis, 2008; Watmough et al., 2009; Kraft et al., 2011; Martínez-Espinosa et al., 2011; Stein, 2011). NORs fall into two different classes: (1) NorVW flavorubredoxin that is employed by many organisms for NO detoxification in response to nitrosative stress from the environment and (2) NORs belonging to the heme-copper oxidase (HCO) superfamily. The enzymes combine two NO molecules to make N_2_O by the input of two electrons. Presently, three types of NORs have been studied in some detail: (1) cNOR (or NorBC) that uses reduced cytochrome *c* as the reductant, (2) quinol-dependent qNOR (qNorB), and (3) qCu_A_NOR that takes both as electron donors. For cNOR and qNOR atomic structures are available (Hino et al., 2012; Matsumoto et al., 2012). Presently, only one qCu_A_NOR has been purified, notably from *Bacillus azotoformans* NCCB 10003 that is closely related to the strain (LMG 9581^T^) discussed here (Suharti et al., 2001, 2004; Lu et al., 2004). The enzyme was reported to be composed of two subunits, a small Cu_A_-type subunit and a large one with two heme *b* molecules. Although quite well characterized, the encoding genes remained elusive.

Besides NORs, the HCO superfamily comprises a broad variety of terminal oxidases (Hemp and Gennis, 2008; Sousa et al., 2012). The common property is a membrane-bound catalytic subunit with 12–14 TMHs that bind a heme *b* (or *a*) for electron transfer and a second heme (*b*_3_, *a*_3_, or *o*_3_) constituting the catalytic center together with an iron (Fe_B_ in NOR) or a copper ion (Cu_B_ in oxidases). Both Fe_B_ and Cu_B_ are ligated by three conserved histidines. Next, two histidines coordinate the electron-transferring heme, whereas one more histidine serves as the proximal ligand to the catalytic heme. This histidine sextet is a signature for HCOs. Oxidases are distinguished by the presence of a tyrosine near the catalytic side that makes a covalent bond with one of the histidines binding Cu_B_. In NORs the pertinent tyrosine is replaced by a glutamate, glutamine, aspartate, or asparagine (Hemp and Gennis, 2008). Moreover, in certain NOR types one of the Cu_B_- or Fe_B_-ligating histidines is substituted by an aspartate (Hemp and Gennis, 2008; Sievert et al., 2008). Besides the catalytic subunit, heme copper oxidases may contain one or more additional subunits for electron transfer as well as membrane-spanning polypeptides for structural integrity. Electron transfer subunits have heme *c* or copper (Cu_A_)-containing cupredoxins as redox components. In addition, heme copper oxidases are distinguished on the basis of their use of the electron donors for O_2_ or NO reduction, which can be either reduced cyt *c* or quinol.

**Figure A2:**
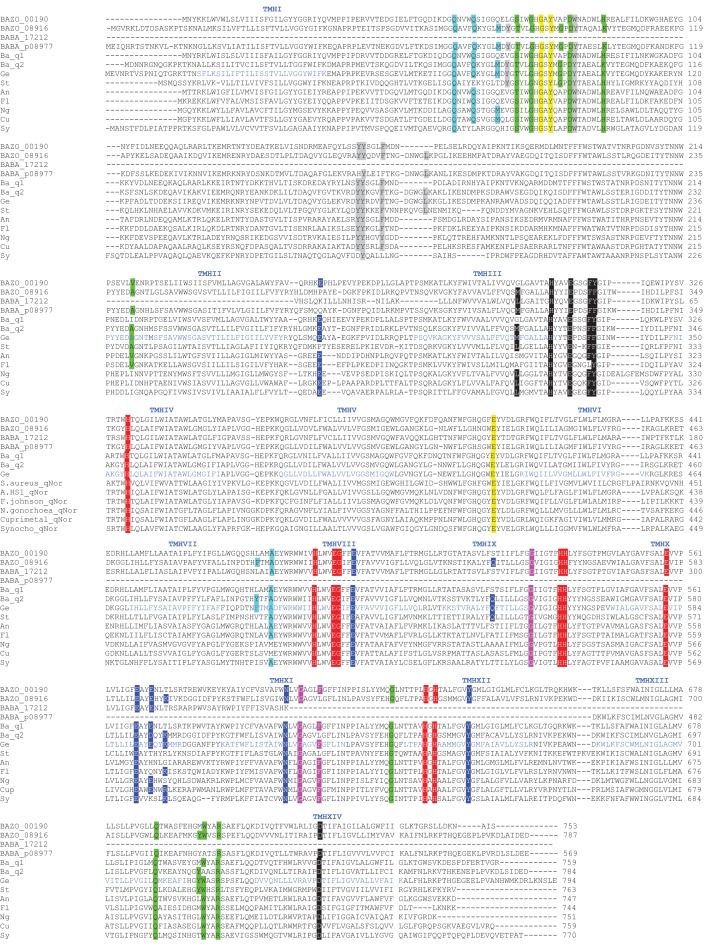
**Multiple sequence alignment of qNOR from *B. azotoformans* LMG 9581^T^, *B. bataviensis* LMG 21833^T^, and other bacteria.** Two non-sense mutations in BABA_p08977 have been corrected in this figure (see text for further explanation). Amino acids involved in the binding of catalytic centers (cyt *b*, *b*_3_, Fe_B_) are highlighted in red, residues for quinol binding in black, bulky residues in the hydrophilic part in gray, residues for Ca^2+^ binding in yellow, residues lining the long water channel from the cytoplasm in dark blue, residues at water channel 1 in green and water channel 2 in light blue; transmembrane regions are indicated in blue and numbered on top of the alignment [as described by Matsumoto et al. (2012)]. Ba_q1, *Bacillus* sp. 1NLA3E (B1NLA3EDRAFT_3340); Ba_q2, *Bacillus* sp. 1NLA3E (B1NLA3EDRAFT_1567); Ge, *Geobacillus stearothermophilus* (PDB 3AYF, 3AYG); St, *Staphylococcus aureus* (SAEMRSA15_02230); An, *Anaerophaga* sp. HS1 (AnHS1_010100003178); Fl, *Flavobacterium johnsoniae* UW101 (Fjoh_2413); Ng, *Neisseria gonorrhoeae* (NgonD_010100007739); Cu, *Cupriavidus metallidurans* CH34 (Rmet_3167); Sy, *Synechocystis* sp. PCC 6803 (sll0450). PDB, protein database accession number.

While cNORs are absent in the genome of *B. azotoformans*, we could identify two genes coding for qNORs, BAZO_00190, and BAZO_08916. The preference for quinol-dependent NOR seems to be a common property of Gram-positive microorganisms. Although BAZO_00190 and BAZO_08916 share only 38% sequence identity, the comparison of their amino acid sequences with those of which the functions have been established, including qNOR from *Geobacillus stearothermophilus* having a known crystal structure (Matsumoto et al., 2012) (Figure A2), suggests functionality of both *B. azotoformans* proteins. Briefly, BAZO_00190 and BAZO_08916 are composed of one subunit in which 13 TMHs surround the catalytic module. A 14th (N-terminal) TMH precedes a soluble domain facing the periplasm with a heme *c* fold, but heme *c* itself is absent. In both proteins amino acids are conserved binding both heme *b* molecules, non-heme iron, a specific calcium atom, the quinol substrate as well as the amino acids lining a proposed water/proton channel down from cytoplasm to the catalytic side, hydrophobic amino acids along two other putative water channels, and aromatic amino acids that stabilize that heme *c* fold of the soluble domain (Figure A2).

cNOR (NorBC) activity depends on ancillary proteins (NorDEFQ) that tend to be encoded on the same operon as the structural proteins (Zumft, 2005b), but qNOR can do without these. Nevertheless, genes *norDQ* (BAZO_01537; BAZO_01542) are present in the genome, although not directly linked to BAZO_00190 and BAZO_08916. This is also the case in genomes of other Gram-positives harboring the *qnorB* gene, including *G. thermodenitrificans* NG80-2, *G. kaustophilus* HTA-426, *Anoxybacillus flavithermus* WK1, *Bacillus licheniformis* ATCC 14580, *Oceanobacillus iheyensis* HTE831, making it a common observation among Gram-positives. However, it is difficult to speculate on their necessity for qNOR functionality in these organisms, as the precise functions of NorD and NorQ remain to be established. In addition, the genome of *B. azotoformans* contains the *norA* gene (BAZO_04395), also termed *dnrA* or *scdA*), which codes for a putative iron-sulfur cluster repair protein in response to NO damage (Overton et al., 2008).

**Figure 2:**
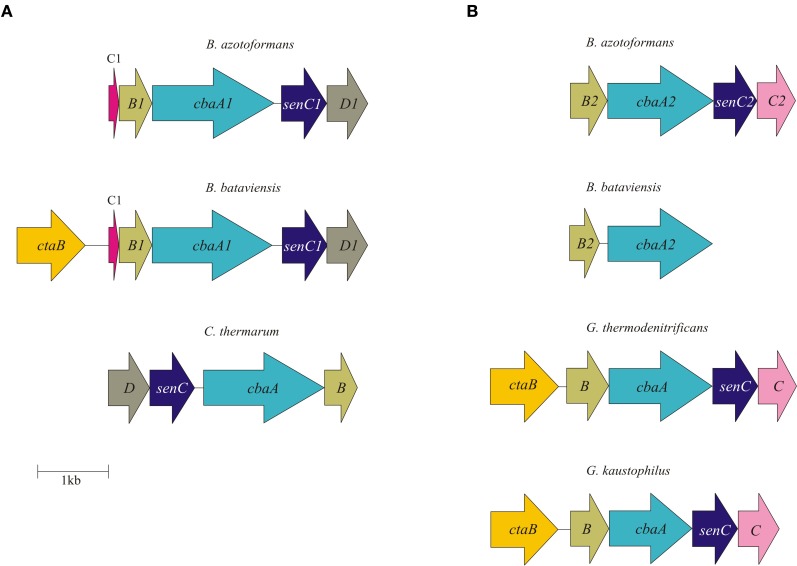
**Physical map of qCu_A_NOR type I (A) and qCu_A_NOR type II (B) gene cluster in *B. azotoformans* LMG 9581^T^, *B. bataviensis* LMG 21833^T^, *Caldalkalibacillus thermarum* TA2.A1, *Geobacillus thermodenitrificans* NG80-2, and *G. kaustophilus* HTA426.** Arrows show the direction of transcription. Open reading frames are drawn to scale. Homologous genes are shown in identical colors.

**Figure A3:**
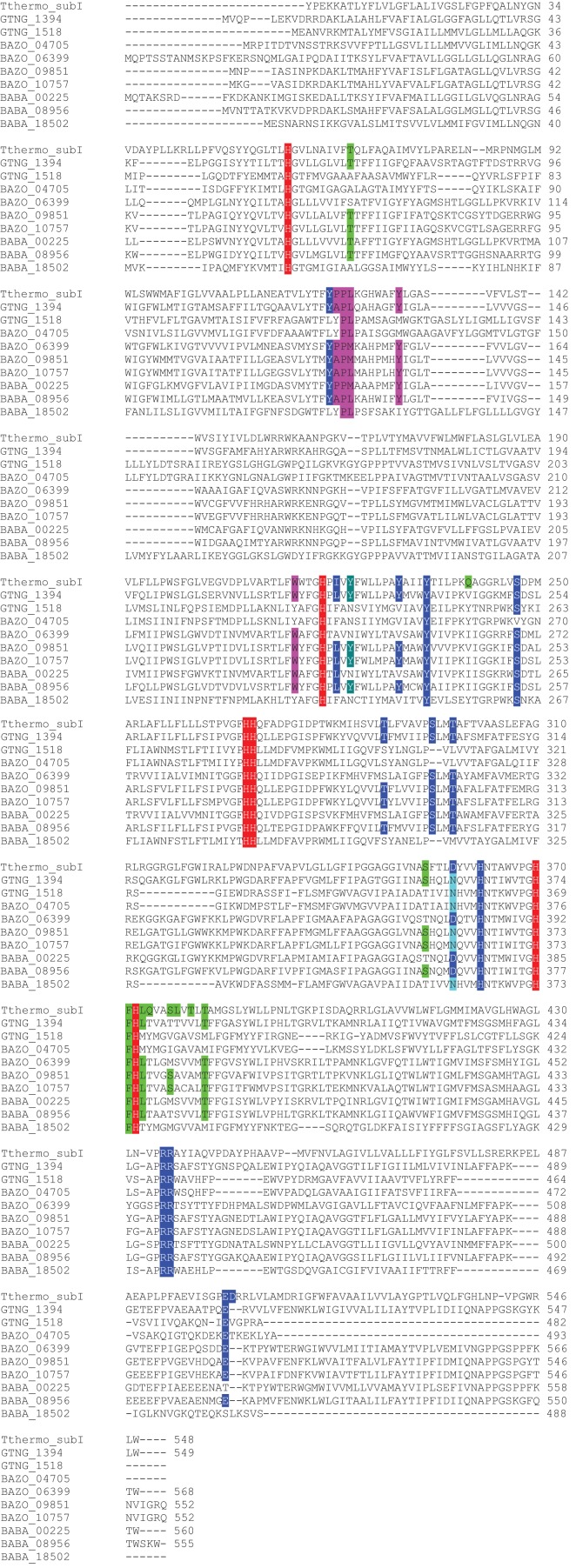
**Multiple sequence alignment of CbaA from *B. azotoformans* LMG 9581^T^, *B. bataviensis* LMG 21833^T^, *Geobacillus thermodenitrificans* NG80-2 (GTNG_1394; GTNG_1518), and. *T. thermophilus* (PDB 3EH3).** Amino acids involved in the binding of heme *b*, heme *b*_3_, and Cu_B_/Fe_B_ are highlighted in red, residues forming D-water channel in blue, residues forming Q-water channel in green, crosslinking tyrosine in oxidases in dark green, residues related with electron transfer in purple. PDB, protein database accession number.

**Figure A4:**
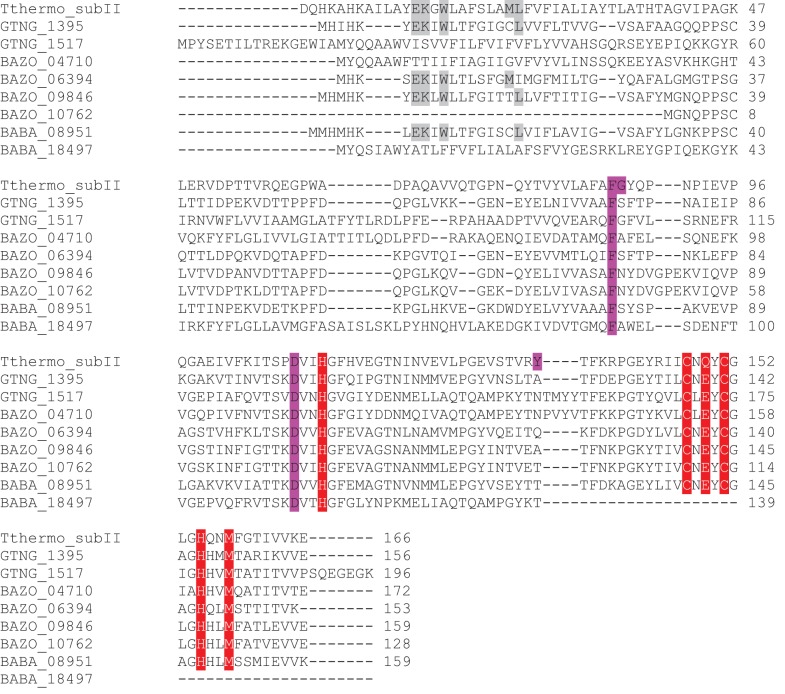
**Multiple sequence alignment of CbaB from *B. azotoformans* LMG 9581^T^, *B. bataviensis* LMG 21833^T^, *Geobacillus thermodenitrificans* NG80-2 (GTNG_1395; GTNG_1517), and *T. thermophilus* (PDB 3EH3).** Contact site residues are highlighted in gray, residues for Cu_A_-binding in red, and residues for electron transport in pink. PDB, protein database accession number.

Besides the two qNORs, no less than four gene clusters are found in the genome of *B. azotoformans* encoding members of the HCO superfamily that share significant sequence homology with cytochrome *c*-dependent *ba*_3_ oxidase (CbaAB) from *Thermus thermophilus* (Figures A3, A4). This is a terminal oxidase that is structurally and functionally quite well investigated [see amongst others: Soulimane et al. (2000); Fee et al. (2008); Smirnova et al. (2008); Liu et al. (2012); von Ballmoos et al. (2012)]. *ba*_3_-type oxidase receives its electrons for O_2_ reduction from cyt *c*. The electrons are transferred via Cu_A_ in subunit II (CbaB), and cyt *b* in subunit I (CbaA) to the *a*_3_-Cu_A_ catalytic center in this subunit. In agreement herewith, all amino acids that had been structurally assigned to Cu_A_ binding and to electron transfer were conserved in the *B. azotoformans* subunits II (Figure A4). Importantly, the N-terminal sequence of BAZO_06394 (MHKSEKIWLTLSFGMIMGFM) is identical to the one of subunit II of qCu_A_NOR published by Suharti et al. (2001). These authors (Suharti et al., 2004) also presented the N-terminal sequence of the large subunit (MTKKNTQEVVKEGREGIGTFIGVGIVGAV), but this was not found in the corresponding subunit I (BAZO_06399). Rather, the almost identical sequence—MATTKNTQEVVKEGREGIGTFIGVGIVGAV—was retrieved in the adjacent gene (contig42_104893_105033) (Figure 2A). This gene encodes a small (46 amino acids) membrane-bound peptide. Hence, it is very well conceivable that this peptide formed part of the enzyme preparation purified by Suharti et al. (2001, 2004). The comparison of the amino acid sequence of subunit I (BAZO_06399) with that of *ba*_3_ from *T. thermophilus* revealed that the tyrosine (Y223, *T. thermophilus* numbering in Figure A3) covalently binding a Cu_B_-associated histidine (H219) was substituted by an asparagine in the *B. azotoformans* protein, in agreement with predictions made by Hemp and Gennis (2008) for NOR functionality (Figure A3). These findings identify BAZO_06394-BAZO_06399 as the dual-function quinol and cyt *c*-dependent qCu_A_NOR described by Suharti et al. (2001, 2004). We may note that the genes coding for these three subunits are linked to two other ones, one (BAZO_06404) coding for a SenC/SCO1-type membrane-bound protein that has been implemented with the insertion of copper (Cu_A_) into subunit I, and BAZO_06409 that is predicted to comprise six TMHs (Figure 2A). In protein databases, homologs of the latter are found being annotated as cyt *c* oxidase-associated membrane proteins. Furthermore, the presence of BAZO_06394-06399 qCu_A_NOR is not restricted to *B. azotoformans* and *B. bataviensis* (see below), but close homologs sharing the same sequential features and the same cluster organization are found in various other Gram-positive bacteria, including *Caldalkalibacillus thermarum* TA2, but also in *Nitrosomonas eutropha* and other nitrifiers (Figure 2A and data not shown). In *N. eutropha* the particular NOR was designated sNOR (Stein et al., 2007). Transcriptome analysis suggested sNOR as a suitable candidate for aerobic NO reduction, next to NorBC (Cho et al., 2006).

*B. azotoformans* likely contains one more novel type of qCu_A_NOR (type II) of which the subunits I and II are encoded by BAZO_04705 and BAZO_04710, respectively (Figure 2B). Again, subunit II is of the Cu_A_-type and in subunit I the cross-linking tyrosine is substituted by an asparagine (Figures A3, A4). At the C-terminal part, the large subunit I is substantially shorter than in the previous case and in *T. thermophilus ba*_3_ oxidase, yet with the conservation of all general HCO characteristics. Being shorter, subunit I is predicted to be structured by 12 TMHs, instead of 13. In contrast, subunit II contains two N-terminal TMHs, one more than in BAZO_06394, and subunit II of *T. thermophilus ba*_3_ oxidase. As above, BAZO_04705-04710 are linked to genes encoding a SenC/SCO1 paralog (BAZO_04700) and a polypeptide (BAZO_04695) with 4–5 predicted TMHs that might constitute a third subunit. Again, the presence of the BAZO_04695-4710 is not restricted to *B. azotoformans*. Close homologs, both at the sequence and cluster organization levels, are found in other Gram-positive bacteria, including *G. thermodenitrificans* NG80-2 (GTNT_1517-1520) and *G. kaustophilus* HTA-426 (GK1670-1673) (Figure 2B and data not shown). Quite remarkably, the gene clusters in both *Geobacillus* species are associated with ones coding for heme *o* oxygenase (GTNG_1516, GK1690). This enzyme converts heme *o* into heme *a*. This might imply that that the *Geobacillus* enzymes would contain heme *a* instead of heme *b*, thereby inventing one more variation on the NOR theme.

The two remaining *ba*_3_ oxidase-related HCO members (BAZO_09851-09846, BAZO_10757-10762) are highly related to one and another. Their large (BAZO_09851, BAZO_10757) and small subunits (BAZO_09846, BAZO_10762) are 83 and 90% identical, respectively. However lacking the first 31 N-terminal amino acids, BAZO_10762 is markedly shorter than BAZO_09846 (128 vs 159 aa). Sequence analyses suggest these HCOs to be genuine (O_2_-reducing) oxidases, having a tyrosine for histidine cross linking. At the sequence levels, the large (I) and small (II) subunits of both *B. azotoformans* proteins are 40 and 45% identical to the ones of *ba*_3_ oxidase from *T. thermophilus* (Figures A3, A4). The differences in amino acid sequences might suggest that the large subunits could bind other hemes and/or differ in the nature of the proton channels. Indeed, sequence identities of BAZO_09851 and BAZO_10757 are significantly higher (67%) with respect to the large subunit of *bo*_3_ cytochrome *c* oxidase isolated from *Geobacillus* (previously *Bacillus*) *stearothermophilus* having heme *o*_3_ at the catalytic side (Sakamoto et al., 1997; Nikaido et al., 1998). The latter protein catalyzes O_2_ reduction with reduced cyt *c*_551_, a lipoprotein, as the specific reductant (*K*_m_ = 0.15 μM). The *bo*_3_ cytochrome *c* oxidase actively pumps protons across the cell membrane during the reaction. It is not known if quinol can substitute for cyt *c*_551_ as electron donor. Also here, the genes coding for both *B. azotoformans* oxidases are linked to ones (BAZO_09841, BAZO_10767) encoding small membrane-bound polypeptides (46 amino acids) possibly serving as a third subunit. We may note that close homologs to BAZO_09851-09841 and BAZO_10757-10767 are present in *G. thermodenitrificans* NG80-2 (GTNT_1394-1396) and *G. kaustophilus* HTA-426 (GK1546-1548), as well as in many other *Bacillus*-related species.

For oxygen respiration, model organism *Bacillus subtilis* strain 168 has the disposal of four different oxidases, cyt *c*_551_-dependent *caa*_3_ oxidase, which is preferentially used at high oxygen concentrations, and three types that rely on quinol: *aa*_3_-600 oxidase, *bd* oxidase, and a *bb*' oxidase (Lauraeus et al., 1991; Azarkina et al., 1999; Winstedt and von Wachenfeldt, 2000). Apart from the *b*(*a/o*)_3_-type oxidase discussed before, which seems to be absent in *B. subtilis*, the genome of *B. azotoformans* has the inventory of two of these. Genes coding for *bd* oxidase are not found in *B. azotoformans*, unlike many other *Bacillus* species including *B. bataviensis* discussed below. Four-subunit quinol-dependent *aa*_3_-600 oxidase encoded by the *qoxBACD* genes are represented by the gene products of BAZO_10131-10146 (subunit II, BAZO_10131; subunit I, BAZO_10136; subunit III, BAZO_10141; subunit IV, BAZO_10146). Amino acid sequences of the *B. azotoformans* gene products readily compare those of established functions. Cyt *c*-dependent *caa*_3_ oxidase is also composed of four subunits (CtaCDEF). The structural genes are usually clustered with genes coding for accessory proteins, including CtaB and CtaA that catalyze the subsequent conversion of heme *b* into heme *o* and heme *a*, and CtaG, a cytochrome *c* oxidase assembly factor. The gene cluster organization (*ctaABCDEFG*) is conserved in *B. azotoformans* (BAZO_04065-BAZO_04035). Furthermore, the individual gene products are well conserved with respect to the ones of which the function has been established. These observations support functionality of both quinol-dependent *aa*_3_-600 (QoxBACD) and of cyt *c*-dependent *caa*_3_ oxidase (CtaCDEF) in *B. azotoformans*.

By exploiting variations on the HCO theme, *B. azotoformans* harbors a repertoire of four different NO reductases and four different oxidases that utilize reduced cyt *c* and/or quinol as reductants. Obviously, this repertoire and the branching in electron transfer may lend the organism the metabolic versatility to cope with large changes in respiratory conditions in the environment.

### Nitrous oxide reduction

The last step in the denitrification pathway to dinitrogen gas is the two-electron reduction of N_2_O to produce, with the input of two protons, N_2_ and water. While the previous steps can be catalyzed by different types of enzymes, only one exists for N_2_O reduction, nitrous oxide reductase (NOS, N_2_OR, NosZ) [see for reviews Zumft (1997, 2005a,b); Zumft and Kroneck (2007)]. NOS is a homodimeric protein with two multinuclear copper centers. In all organisms investigated thus far, it is located in the periplasm and in Gram-negative species it is a soluble protein. For its assembly, NOS depends on a number of accessory proteins (NosADLYF). Assembly is thought to take place in the cytoplasm after which NOS is exported using the TAT translocon. In Gram-negative bacteria, the TAT signal sequence is unusual long counting approximately fifty amino acids.

**Figure 3:**
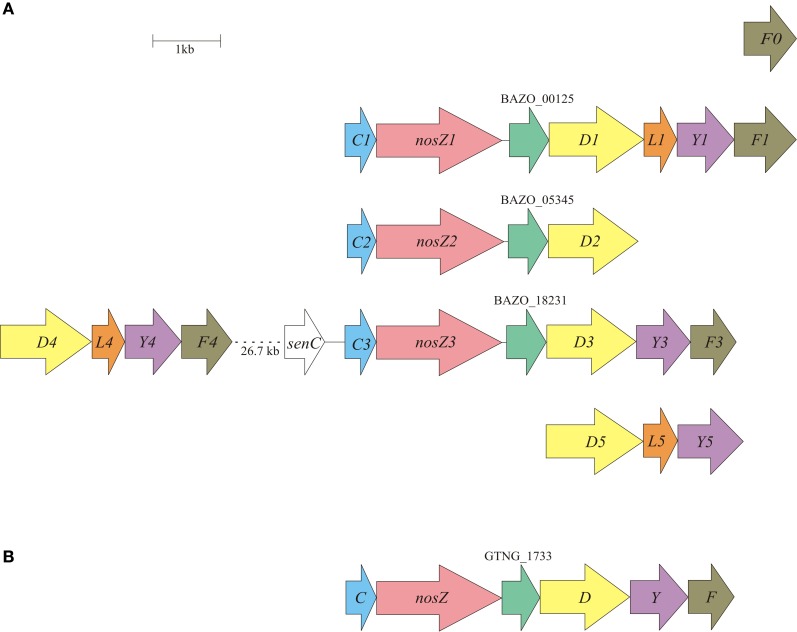
**Physical map of the *B. azotoformans* LMG 9581^T^ (A) and *Geobacillus thermodenitrificans* NG80-2 (B) *nos* gene clusters.** Arrows show the direction of transcription. Open reading frames are drawn to scale. Homologous genes are shown in identical colors.

The genome of *B. azotoformans* encodes no less than five *nos* gene clusters, with three clusters including a *nosZ* gene, and one orphan *nosF* (Table 2, Figure 3A). Gene arrangements differ in every cluster (Figure 3A), but they are most similar to the atypical *nos* gene cluster (*nosCZ*-ORF-*nosDYF*-ORF) found in the three other Gram-positives available in the databases with *nos* genes: *Geobacillus thermodenitrificans* NG80-2 (Figure 3B), *Desulfitobacterium hafniense* T51 (Liu et al., 2008), and *Desulfotomaculum ruminis* DSM 2154. Each *nosZ* gene is consistently preceded by *nosC*, coding for a cytochrome *c*, which is predicted (LipoP) to be a lipoprotein with the heme *c* part facing the periplasm. *NosZ* is followed by a conserved hypothetical open reading frame encoding a membrane protein with four predicted TMHs. Interestingly, the latter is also the case in *W. succinogenes* (Ws0918) (Simon et al., 2004). In *W. succinogenes*, the two copies of *nosC* are located downstream of *nosZ* and are flanked by *nosG* and *nosH* genes, which are absent in *B. azotoformans.* NosGH represent a membrane-bound quinone-oxidizing electron transfer module, resembling NapGH in the periplasmic NAR (Nap) system described above. *B. azotoformans* also lacks a *nosA* gene that codes for a periplasmic accessory protein involved in Cu binding. However, we note the presence of one of the four *senC/SCO1* paralogs upstream of *nosC*3. As mentioned before, SenC has been implemented with the insertion of copper (Cu_A_); genes coding for the other three paralogs are associated with the alternative NORs. NosL, which is present in three copies in the *nos* gene clusters (Figure 3A), is a lipoprotein that stoichiometrically binds Cu. This property suggests NosL to be a copper chaperone for metal-center assembly (Zumft, 2005a). NosDYF represent a Cu-ABC transport system with a periplasmic Cu-binding protein (NosD), a membrane-bound permease (NosY), and a cytoplasmic ATPase (NosF) that provides the driving force for Cu translocation (Zumft, 2005a). As can be seen from Figure 3A, *nosDYF* come in different arrangements in the genome of *B. azotoformans*, either with or not associated with *NosZ*. Moreover, sequence analysis reveals marked differences among the individual gene products. All five *nosD* gene products have a putative transmembrane helix at the C-terminus, but only three of these have a predicted Sec-signal (NosD1, BAZO_00130; NosD4, BAZO_18066; NosD5, BAZO_14249), as expected for periplasmic proteins. NosY3 contains a seventh TMH, one more than usual, while significant insertions without any known domain are found in NosY4 (39 AA at the N-terminus) and NosF1 (49 AA at the C-terminus). Regardless of these differences, *B. azotoformans* has the disposal of a multicopy factory that could supply NosZ, and possibly also other copper proteins like NirK, with the high demand on copper (Richardson et al., 2009).

**Figure A5:**
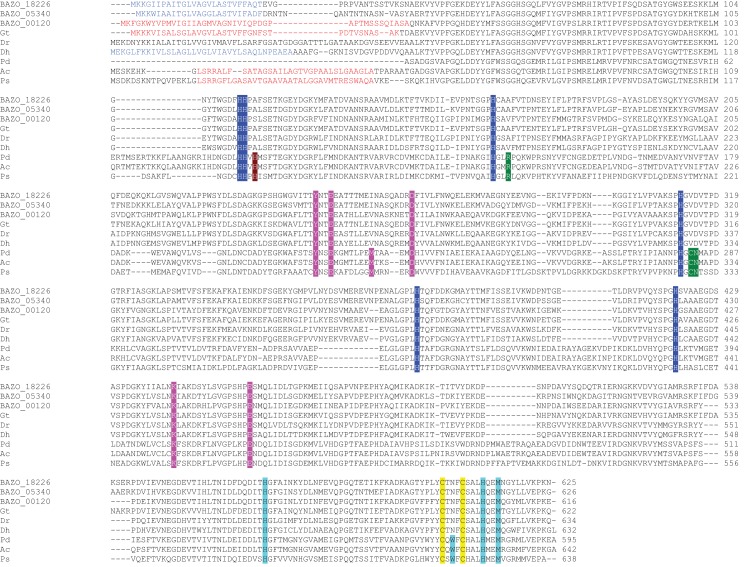
**A Multiple sequence alignment of NosZ from *B. azotoformans* LMG 9581^T^ and other bacteria.** Conserved histidine ligands of the Cu_Z_ center are highlighted in dark blue; cysteine and other ligands of the Cu_A_ center in yellow, and turquoise respectively; partially conserved histidine residues in brown; calcium atom and chloride ion ligands in pink and green, respectively [according to Simon et al. (2004)]. TAT sequences are printed in red, N-terminal cleavage in blue. Gt, *Geobacillus thermodenitrificans* NG80-2 (GTNG_1734); Dh, *Desulfitobacterium hafniense* T51 (DSY0261); Dr, *Desulfotomaculum. ruminis* DSM 2154 (Desru_1528), Pd, *Paracoccus denitrificans* DSM 413 (PDB 1FWX), Ac, *Achromobacter cycloclastes* 1013 (PDB 21WF); Ps, *Pseudomonas stutzeri* (PDB 3SBP). PDB, protein database accession number.

The deduced primary structure of the three *B. azotoformans* NosZ proteins (between 76.5 and 83.1% sequence identity) show all conserved ligands of Cu_A_ and Cu_*Z*_ centers that have been identified in the crystal structures (Figure A5) (Brown et al., 2000; Paraskevopoulos et al., 2006). Nevertheless, the NosZ sequences contain insertions and deletions that are shared with nitrous oxide reductases from other Gram-positive species, likely placing these in a distinct family. The differences could be related with domain-specific interactions in Gram-positives and Gram-negatives with other components of the NosZ system. Another feature is that the N-terminal sequence in Gram-positive NosZ's is shorter (Figure A5). Moreover, SignalP and TatP prediction programs are somewhat ambiguous regarding the presence of N-terminal leader and TAT signal sequences. For instance, whereas NosZ1 from *B. azotoformans* is predicted to have a TAT leader, yet lacking an N-terminal cleavage side, the opposite holds for NosZ2 and NosZ3. These differences might point to, even protein-specific, differences in assembly, and transport. It is conceivable that transport proceeds not only by the TAT—but also by the Sec translocon. Indeed, it has been described for *W. succinogenes* that transport of non-folded NosZ and subsequent Cu insertion results in a fully functional protein (Heikkilä et al., 2001).

NosZ from Gram-negative bacteria is, as mentioned, a soluble protein in the periplasm where it receives its electrons for N_2_O reduction from reduced cytochrome *c*. In Gram-positive microorganisms, including *B. azotoformans* strain NCCB 10003, nitrous oxide reductase activity appears to be associated with the membrane fraction only [(Suharti and de Vries, 2005), and references herein]. Considering that the *B. azotoformans* NosZ gene products do not possess any TMH other than an N-terminal Sec-or TAT-signal, the conserved ORF encoding the polypeptide with four TMHs might provide a platform for membrane association. In addition, *B. azotoformans* nitrous oxide reductase(s) employ both cyt *c* and menaquinol as electron donors (Suharti and de Vries, 2005). Clearly, the particular lipoprotein cytochromes *c* that are encoded by the *nosC* genes might assist in cyt *c*-dependent electron transfer, but the role of menaquinol is more puzzling. Menaquinol could act directly or indirectly, viz. via menaquinol: cytochrome *c* oxidoreductase (*bc*_1_/*b*_6_*f* complex; see hereafter). A more direct role presumes the presence of a membrane-bound quinol oxidase in contact with NosZ. Here, the conserved membrane protein would come to mind. Alternatively, NapGH might serve such function. Experimental research has to decide between these or other alternatives.

### *bc*_1_/*b*_6_*f* complex of *B. azotoformans*

In nitrate/nitrite-respiring Gram-negative bacteria, (mena)quinol: cytochrome *c* oxidoreductase (*bc*_1_ complex) plays a central role in the energy metabolism. By the complex, quinol that is produced concomitant to the oxidation of (organic) substrates is re-oxidized with cyt *c* as electron acceptor. Taking advantage of the proton-motive Q cycle, quinol:cyt *c* oxidoreduction is associated with the pumping of protons (4H^+^/2e^−^) across the cell membrane. Reduced cyt *c* serves as the reductant of the nitrate-reducing steps that, in case of a localization in the periplasm, do not contribute to *pmf* generation themselves. The work by the group of de Vries (Suharti et al., 2004, Suharti and de Vries, 2005) demonstrates that *B. azotoformans* opts for branched electron transfer, using both quinols and reduced cyt *c* for denitrification (see also above). While the cyt *c*-dependent pathway is energetically more favorable, a direct coupling of the denitrification steps with quinol oxidation could be kinetically more attractive (Suharti et al., 2004; Suharti and de Vries, 2005). The former pathway calls for the presence of a *bc*_1_/*b*_6_*f* complex in *B. azotoformans*, as well as suitable cytochromes *c* for electron transfer.

In its simple, canonical form the *bc*_1_ complex comprises three subunits: a Rieske-type 2Fe-2S protein and a membrane protein that binds one heme *b* and one quinone at the cytoplasmic face and another heme *b* and a quinone near the periplasm. The third component is cyt *c*. In *Bacillus subtilis*, the organization of the complex is different, resembling *b*_6_*f* complexes of *Cyanobacteria* and chloroplasts (Yu et al., 1995; Yu and Le Brun, 1998). The Rieske iron sulfur protein (QcrA) is of a general type, but the cytochrome *b* subunit is split into two parts (QcrA and QcrB). QcrB is homologous to the N-terminal part and QcrC is to the C-terminal part of common cytochrome *b* and of subunit IV of the *b*_6_*f* complex. At its N-terminus, QcrC is fused to a *c*-type cytochrome localized in the periplasm. The particular organization and the related amino acid sequences are conserved in QcrA (BAZO_09251), QcrB (BAZO_09246), and QcrC (BAZO_09241) from *B. azotoformans* (data not shown). We note the presence in the *qcrABC* gene cluster of a set of flanking genes coding for membrane-bound proteins (BAZO_09231; BAZO_09236; BAZO_09261) and for proteins with tetratrico peptide repeats (TPR) (BAZO_09256; BAZO_09266). Their presence is conserved in many *Bacillus* species, including *B. bataviensis*. This observation might suggest *b*_6_*f* of these organisms to be more “complex.”

For electron transfer, *B. subtilis* has the disposal of only two small membrane-bound *c*-type cytochromes: CccB, a cyt *c*_551_ lipoprotein, and CccA, a cyt *c*_550_ that is anchored to the membrane by a single C-terminal TMH (Bengtsson et al., 1999). De Vries and coworkers (Suharti et al., 2004; Suharti and de Vries, 2005) were able to isolate and characterize three different types of monoheme cytochrome *c*-type lipoproteins from *B. azotoformans*, namely cyt *c*_550_, cyt *c*_551_, and cyt *c*_552_; their encoding genes were not identified. All three could be reduced in concert with menaquinol oxidation by the *b*_6_*f* complex. Cyt *c*_551_ functioned as the specific electron donor to qCu_A_NOR, but it was inactive in nitrite and in nitrous oxide reduction. In addition, pseudoazurin or other cupredoxins, the principal electron donors for copper-containing NirK, appeared to be absent (Suharti and de Vries, 2005). Now looking at the genome of *B. azotoformans*, we note the presence of four genes coding for monoheme cyt *c*-type lipoproteins, three of which being linked to the *nosZ* genes (see above). This would leave the gene product of the fourth candidate (BAZO_07449)—indeed annotated as cyt *c*_551_—as the electron carrier for qCu_A_NOR. Furthermore, diheme cyt *c*_5_ is specifically required for NirK activity in *Neisseria meningitides* (Deeudom et al., 2008). The genome of *B. azotoformans* houses only one gene encoding a diheme cytochrome *c* lipoprotein (BAZO_12419) that consequently might serve nitrite reduction by NirK in this organism.

### Gene inventory of *B. bataviensis*

*B. bataviensis* is capable of denitrification, albeit not to N_2_ but with the stoichiometric production of N_2_O from nitrate [(Verbaendert et al., 2011); Verbaendert et al., unpublished data]. This presumes the presence of the complete denitrification pathway lacking nitrous oxide reductase (NosZ). A first glance at the genome of *B. bataviensis* shows this to be the case. NosZ and its accessory proteins are absent, but the gene inventory for reduction of nitrate to N_2_O is available (Table 3). The more close inspection reveals a more complicated situation with sets of truncated or degenerated (pseudo)genes that are difficult to reconcile with full functionality of their gene products. In addition, we note quite interesting differences in the denitrification potentials between *B. bataviensis* and *B. azotoformans*.

For cytoplasmic dissimilatory nitrate reduction to nitrite, the genome of *B. bataviensis* contains one *narGHJI* gene cluster (BABA_18582-18597) with two linked *NarK* copies (BABA_18532, BABA_18537) in close vicinity (Figure 1A). The NarG, H, J, and I proteins display all conserved features, indicating that NAR is the active NAR in *B. bataviensis*. This has to be the case since the organism is devoid of the alternative, periplasmic NAR system (NAP). Indeed, none of the *nap* genes are found in the genome, except a putative *napH* (BABA_14547). Its gene product (153 aa) is significantly shorter than NapH (~300 AA) with an established function (Simon et al., 2003; Kern and Simon, 2008), thereby lacking all cysteines for the binding of iron sulfur clusters. Rather, InterProScan suggests BABA_14547 to be an FMN-binding protein.

For dissimilatory reduction of nitrite to NO, again only one candidate is present, a *nirK* gene that is designated as a pseudogene (BABA_p06582) by the presence of an apparent interspersing stop codon. However, by the correction for a single non-sense mutation (A–C at position 38028 of contig 42), the DNA sequence translates into an amino acid sequence that is 79.8% identical to NirK from *B. azotoformans*, differing only three amino acids in length. Moreover, by this substitution all features would be conserved that are related with the binding of the catalytic copper atoms and with the characteristic secondary structural elements of NirK2 family proteins (see above). Again bearing in mind that *B. bataviensis* is capable of nitrite reduction to NO, we anticipate that this pseudogene, caused by a single non-sense mutation, is probably the result of a sequencing artifact (although base calling was unambiguous). Proteome analysis should confirm this expectation. Like *B. azotoformans, B. bataviensis* is proposed to carry out also dissimilatory nitrate reduction to ammonia (Table 3). The *nrfHA* gene products (BAZO_17652-17657) meet all criteria described above for functionality (see Figure A1). The question is under which conditions the genes are expressed, taking into account that during standard denitrifying growth nitrate is quantitatively reduced to N_2_O.

Contrary to *B. azotoformans*, *B. bataviensis* seems to have the genetic potential for assimilatory nitrate and nitrite reduction to ammonium. The gene inventory is made up by *nasC* (BABA_16882) and *nirDB* (*nasGB*) (BABA_00910-00915) coding for assimilatory nitrate and nitrite reductase, respectively. Mechanistically, the process could be somewhat different from the one investigated thus far. In many microorganims, *nasC* and *nirDB* (*nasGB*) are localized on the same operon together with NarK-type transporters and regulatory proteins (Luque-Almagro et al., 2011). In *P. denitrificans* NasC and NasGB presumably function as one protein complex (Gates et al., 2011). Herein, NasB (NirB) binds NADH that serves as the reductant for both the six-electron reduction of nitrite to ammonia (by NasB) and the two-electron reduction of nitrate to nitrite (by NasC). The Rieske-type 2Fe-2S protein NasG (NirD) intermediates electron transfer between both catalytic subunits. In *B. bataviensis nasC* and *nirBD* are found in different parts of the genome and are not linked to NarK-type transporters. We note, however, the presence in the *nirBD* cluster of three genes (BABA_00890-00905) encoding key enzymes for the synthesis of sirohydrochlorin, the prosthetic group of NirB. NasC and NirB are highly similar to the respective proteins of which the function has been established, albeit with a notable difference. NasC (729 AA) from *B. bataviensis* is shorter than the protein from, for instance, *P. denitrificans* (870 AA) and it lacks at its C-terminal part a number of cysteines that might bind an iron-sulfur cluster. However, this difference is shared with many other Gram-positive microorganisms. Although *B. bataviensis* NirD is 44–57% identical to NirDs from other *Bacillus*-related species, there is one important distinction: in the former two key amino acids (cysteine, histidine) are substituted that are involved in the binding of the 2Fe-2S cluster. In contrast, *nasC* is linked with two genes coding for a hybrid iron sulfur protein (HCP) (BABA_16877) and for another Rieske-type 2Fe-2S protein (BABA_16872). The particular properties are consistently shared with other species, like *Bacillus* sp. 1NLA3E, suggesting an alternative, yet functional assimilatory nitrate/nitrite reductase system in *B. bataviensis*. For the uptake of nitrate, the genome of *B. bataviensis* contains four *narK* copies, unlike *B. azotoformans* that has only one. Two of these (NarK2, BABA18537; NarK3, BABA_18532) were already encountered in connection with dissimilatory NAR. They belong to the NarK2 family of nitrate:nitrite antiporters, facilitating the uptake of nitrate and the export of toxic nitrite. NarK1 (BABA_15587) and NarK4 (BABA_18532) are affiliated with the NarK1 family of (high-affinity) nitrate:proton symporters, which would make these proteins attractive candidates to serve N-assimilation. In contrast again to *B. azotoformans*, a gene is present in the genome of *B. bataviensis* (BABA_17227) coding for an AmtB-type of ammonium transporter.

Regarding NO reduction to N_2_O and the role therein of HCO proteins, the genome picture is ambiguous. *B. bataviensis* contains two copies of *qnorB* and three HCO copies related to cytochrome *c*-dependent *ba*_3_ oxidase (*cbaAB*) from *Thermus thermophilus* (Table 3). One *qnorB* is a pseudogene (BABA_p08977), with two frame shifts, either resulting from actual point mutations or sequencing errors (although base calling was unambiguous). When the gene sequence was altered manually at both positions (changing T into C at position contig55_76292 and A into G at position contig55_76206), the resulting amino acid sequence became highly similar to qNorB sequences from *Geobacillus* and *Bacillus* strains (AA sequence identity, 79–83%). However, it still contained a 217-AA deletion near the C-terminus (from position 464 to 760 in *G. thermodenitrificans* numbering; see Figure A2) comprising only 8 out of 14 TMHs and lacking five out of six conserved histidine residues. Also the other *qnorB* gene (BABA_17212) is truncated. It is devoid of 273 N-terminal and approximately 150 C-terminal amino acids, again spanning only 8 TMHs. Thus, it seems that both qNorBs are corrupted and inactive. Sequence analysis addresses two of the three *ba*_3_-type oxidases (CbaAB) (BABA_00225-00230, BABA_18502-18497) to the alternative NORs (Figures A3, A4). However, subunit II of the latter one (BABA_18497) lacks approximately 30 amino acids at its C-terminus, including the ones involved in binding of Cu_A_. In addition, BABA_18502-18497 are not linked to genes coding for the proposed membrane-bound subunit III and for a SenC/SCO1 protein for Cu_A_ insertion. These observations would overrule a role for cyt *c* as an electron donor, although a function as such for quinol is still feasible. Subunits I (BABA_00225) and II (BABA_00230) of the second candidate are 70 and 79% identical to the respective subunits of BAZO_06399-006394 that has been identified above as qCu_A_NOR (sNOR) (Figures 2, A3, A4). Furthermore, the N terminus of subunit II is quite similar to the one determined by Suharti et al. (2004), having only three mismatches. In full agreement with BAZO_06399-06394, the genes of the *B. bataviensis* are linked to ones encoding a small membrane-bound polypeptide (46 amino acids), another membrane protein (BABA_00215) with 6 TMHs and a SenC/SCO1-type protein (BABA_00220) (Table 3; Figure 2). Unlike BAZO_06399-006394 and all its other relatives, the *B. bataviensis* gene cluster is associated with a gene coding for heme *o* synthetase (*ctaB*; BABA_00235). This might imply that the particular *B. bataviensis* HCO contains cyt *o*_3_ at its catalytic side. Nevertheless, the striking resemblance regarding amino acid sequences and the organization of the gene cluster highly suggest BABA_00225-00230 and its associated small membrane subunit to be a menaquinol/cyt *c*-dependent NOR.

The third *ba*_3_-type (CbaAB) HCO (BABA_08951-08956) is most likely an oxidase, as deduced from its amino acid sequence having the crosslinking tyrosine (Figure A3). It is one of the four oxidases that are detected in the genome. Like in *B. azotoformans*, oxygen respiration may be mediated by quinol-dependent dependent *aa*_3_-600 oxidase encoded by the *qoxBACD* genes (BABA_05106-05091) and by cyt *c*-dependent *caa*_3_ oxidase (CtaCDEF). In agreement with other organisms, structural genes form part of a larger cluster (*ctaABCDEFG*) (BABA_16147-16177) that includes the *ctaA* and *ctaB* genes encoding cyt *a* and cyt *o* biosynthesis proteins. A sequence comparison of the gene products just mentioned with those of verified proteins indicates *aa*_3_-600 oxidase and cyt *c*-dependent *caa*_3_ oxidase to be functional (data not shown). Besides these three, we find conserved genes encoding the alternative quinol-dependent cytochrome *bd* oxidase (CydAB, BABA_01890-01895). CydAB is an oxidase that is found in many microorganisms and that, because of its high affinity for oxygen, (Borisov et al., 2011) is expressed under O_2_ limitation. *B. azotoformans* does not contain this oxidase. By the availability of the four different oxidases, *B. bataviensis* would have the potential to aerobically respire under a wide range of oxygen concentrations. However, the organism seems to be more limited in nitrate respiration as compared to *B. azotoformans.*

## Concluding remarks

**Figure 4:**
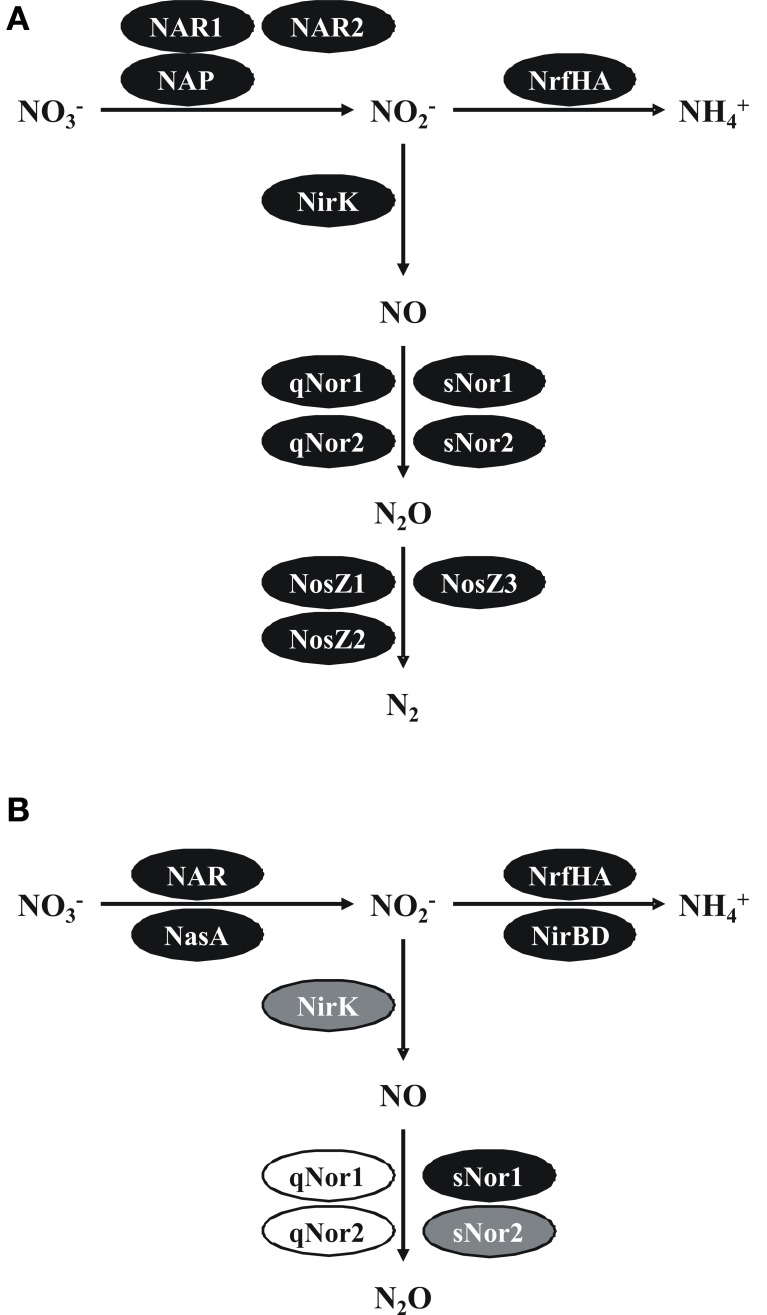
**Pathways and functional gene inventories for denitrification and ammonification in *B. azotoformans* LMG 9581^T^ (A) and *B. bataviensis* LMG 21833^T^ (B).** Black ovals indicate predicted functional enzymes, gray ovals likely functional enzymes and white ovals pseudogenes or corrupted enzymes. NAR, cytoplasmic dissimilatory nitrate reductase; NAP, periplasmic dissimilatory nitrate reductase; NasC, assimilatory nitrate reductase; NrfHA, dissimilatory nitrite reductase to ammonium; NirBD, assimilatory nitrite reductase; NirK, periplasmic nitrite reductase to nitric oxide; qNor, dissimilatory quinol-dependent nitric oxide reductase; sNor, dissimilatory menaquinol/cyt *c*-dependent nitric oxide reductase; NosZ, dissimilatory nitrous oxide reductase. See text for more explanations.

Above, we made a comprehensive analysis of the functional denitrification gene inventory of two *Bacillus* species. Our results are schematically presented in Figure 4. Our exploration came with quite some surprises, we detected potential new enzymes, supported previous suggestions and raised interesting questions that need experimental answers, which we briefly would like to summarize.

A first surprise was that *B. azotoformans*, a well-known denitrifier, and *B. bataviensis* that is less well understood in this respect, are both capable of denitrification and dissimilatory ammonification (DNRA). Especially, *B. azotoformans* that makes N_2_ as the end product of nitrate denitrification has a large arsenal of apparently redundant enzymes, but that most likely would enable the organism to thrive under highly variable environmental conditions. The N_2_O-producing *B. bataviensis* is much more restricted in this. We note the presence of various pseudogenes that could be relicts of earlier times or situations in which this microorganism could be more dependent on anaerobic nitrate respiration. Striking is the way both *Bacillus* species took advantage of the opportunities of heme copper oxidases for NO and O_2_ reduction. Our analysis permitted the identification of the genes coding for bi-functional qCu_A_NOR purified and characterized before Suharti et al. (2001, 2004). Yet, we could detect other related novel enzymes that either reduce NO or O_2_, but that still need to be validated by biochemical research.

Gram-positive microorganisms typically have limited space in their periplasm, which might pose specific demands on the way denitrification processes are structured in the periplasm. Suharti and de Vries (2005) already noticed that virtually all denitrifying partial reactions in *B. azotoformans* strain NCCB 10003 were associated with the cell membrane. Our genome analyses support their observations. All enzymes appear to be membrane-bound, either by TMHs at catalytic enzymes themselves, through association with membrane-bound partners or by covalent binding to lipids (NirK, NosZ). Membrane association most likely requires specific structural and architectural adaptations that are reflected in amino acid sequences. In agreement herewith, we note that the denitrification enzymes discussed often form part of (sub)families that are specific for Gram-positive microorganisms. Another aspect that relates to the membrane-bound character is energy metabolism. As pointed out again by De Vries and his coworkers (Suharti et al., 2004; Suharti and de Vries, 2005), all reactions tested used menaquinol as the reductant. Our analyses confirm these findings. However, also cyt *c* could be utilized as the reductant. This branched electron transfer adds to the metabolic flexibility. The use of (mena)quinol may allow reactions to proceed at high rates, also under conditions of substrate limitation. The use of reduced cytochrome(s) produced through quinol oxidation by the *bc*_1_/*b*_6_*f* complex is energetically more favorable.

All in all, our analyses suggest an astonishing versatility in denitrification opportunities, especially for *B. azotoformans*. The important question is, if and under which environmental conditions the different (partial) processes are expressed. An answer to this needs experimental research.

### Conflict of interest statement

The authors declare that the research was conducted in the absence of any commercial or financial relationships that could be construed as a potential conflict of interest.
